# Changes in the utilisation of acute hospital care in Ireland during the first wave of the COVID-19 pandemic in 2020

**DOI:** 10.12688/hrbopenres.13307.3

**Published:** 2022-10-12

**Authors:** Louise Marron, Sara Burke, Paul Kavanagh

**Affiliations:** 1 Department of Public Health HSE East, Dr Steevens’ Hospital, Dublin 8, Ireland; 2 Centre for Health Policy and Management, Trinity College Dublin, Dublin 2, Ireland; 3 Health Intelligence Unit, Strategic Planning and Transformation, Jervis House, Jervis St, Dublin 1, Ireland; 4 Department of Epidemiology and Public Health, Royal College of Surgeons, Dublin 2, Ireland

**Keywords:** COVID-19, health systems, health services, secondary care, healthcare utilisation, public health, mental health, alcohol

## Abstract

Background: Reduced and delayed presentations for non-COVID-19 illness during the COVID-19 pandemic have implications for population health and health systems. The aim of this study is to quantify and characterise changes in acute hospital healthcare utilisation in Ireland during the first wave of COVID-19 to inform healthcare system planning and recovery.

Methods: A retrospective, population-based, observational study was conducted using two national datasets, Patient Experience Time (PET) and Hospital In-Patient Enquiry (HIPE). The study period was 6th January to 5th July 2020.

Results: Comparison between time periods pre- and post-onset of the COVID-19 pandemic within 2020 showed there were 81,712 fewer Emergency Department (ED) presentations (-18.8%), 19,692 fewer admissions from ED (-17.4%) and 210,357 fewer non-COVID-19 hospital admissions (-35.0%) than expected based on pre-COVID-19 activity. Reductions were greatest at the peak of population-level restrictions, at extremes of age and for elective admissions. In the period immediately following the first wave, acute hospital healthcare utilisation remained below pre-COVID-19 levels, however, there were increases in emergency alcohol-related admissions (Rate Ratio 1.22, 95% CI 1.03, 1.43, p-value 0.016), admissions with self-harm (Rate Ratio 1.39, 95% CI 1.01, 1.91, p-value 0.043) and mental health admissions (Rate Ratio 1.28, 95% CI 1.03, 1.60, p-value 0.028).

Discussion: While public health implications of delayed and lost care will only become fully apparent over time, recovery planning must begin immediately. In the short-term, backlogs in care need to be managed and population health impacts of COVID-19 and associated restrictions, particularly in relation to mental health and alcohol, need to be addressed through strong public health and health system responses. In the long-term, COVID-19 highlights health system weakness and is an opportunity to progress health system reform to deliver a universal, high-quality, sustainable and resilient health system, capable of meeting population health needs and responding to future pandemics.

## Introduction

The coronavirus disease 2019 (COVID-19) pandemic, caused by severe acute respiratory syndrome coronavirus 2 (SARS-CoV-2), presents a significant challenge to national health systems across the globe. In addition to controlling the transmission of infection across the population and ensuring sufficiency of health services to meet demand, impacts on the provision of non-COVID-19 care are reported in many countries ^1, 2^. Internationally, reduced and delayed presentations for non-COVID-19 illness are linked to increased morbidity and mortality ^3– 5^. These changes in utilisation of healthcare have public health implications for both population health and health systems in the short term and beyond ^2^.

Ireland has faced these direct and indirect impacts of COVID-19 from a unique position. A decade ago, the Irish health system experienced severe cutbacks during a prolonged period of financial austerity ^6^. Since 2017, a significant programme of reform entitled Sláintecare has been adopted by government ^7– 9^. Sláintecare is a ten-year plan for systemic health reform which seeks to tackle long-recognised health system capacity deficits and fragmentation, Ireland’s over-reliance on acute hospital services, poor orientation to primary, community care services and public health, underpinned by the absence of universal access to health and social care ^7, 10^.

The aim of this study is to describe and quantify the impact of the first wave of the COVID-19 pandemic on acute healthcare utilisation in Ireland in order to inform healthcare system planning and public health policy. This work is situated within a broader research project which is co-producing research and evidence to inform health system and policy decisions ^11^. The data and analysis presented here is part of the Foundations’ Living Implementation Framework with Evaluation (LIFE) ^11^.

## Methods

### Study design and setting

A retrospective, population-based, observational study was conducted to quantify and characterise acute public hospital service utilisation events in Ireland and to compare these events across different time periods with reference to the epidemiology and public health management of COVID-19. Emergency Department (ED) presentations, admissions from ED and non-COVID-19 in-patient admissions to Health Service Executive (HSE) acute public hospitals over a 26-week period from 6 ^th^ January 2020 to 5 ^th^ July 2020 were identified, analysed and compared with those observed over defined reference periods.

### Data sources

***Patient Experience Time (PET).*** National data ED attendances were obtained from the Patient Experience Time (PET) dataset which is an administrative dataset that contains observations of individual-level ED utilisation across 30 HSE-operated or funded hospitals ^12^. PET contains information on age, sex, discharge destination, mode of arrival and referral and triage status. Clinical information is not reported and therefore patients with and without COVID-19 were included in the data used for this study. PET data does not include Minor Injury Units (MIU), private EDs, specialist EDs or direct attendance at acute assessment units.

***Hospital In-Patient Enquiry (HIPE).*** National acute hospitals discharge data were accessed from the Hospital In-Patient Enquiry (HIPE) data via the Health Intelligence Unit (HIU) Health Atlas Ireland Analyser. HIPE is managed by the Healthcare Pricing Office (HPO) and is a well-established, quality-assured health information system that is the primary source of episode-based, aggregate clinical, demographic and administrative data on discharges from acute public hospitals in Ireland ^13^. It contains information on age, sex, area of residence, admission type, date of admission and discharge along with principal diagnosis coded using the International Classification of Diseases Tenth Revision (ICD-10) ^14, 15^. It is used nationally to inform healthcare planning, management and activity-based funding ^16^.

### Variables

***Exposure.*** The exposure was to COVID-19 and the associated public health restrictions and wider socioeconomic changes within 2020. The study period was divided into four sub-periods ( Table 1). These time periods reflect levels of exposure based on the *a priori* knowledge of the epidemiology of COVID-19 during the first wave, and of the public health measures implemented. Period 1 was defined as prior to the beginning of the first wave, Periods 2 and 3 were periods where progressive public health restrictions were implemented and Period 4 commenced with the easing of public health restrictions. Period 1 was defined as starting on the first Monday of January for the study period and reference periods, which were divided into the same sub-periods. The historic reference period for the PET data was a 26-week time period beginning on the first Monday of January 2019. This dataset has increased in completeness year-on-year so restricting the reference period to 2019 allowed meaningful comparison. The historic reference period for the HIPE data was a 26-week time period beginning on the first Monday in January for 2017–2019. It was assumed that while there might be a slight variation year on year, the three-year average of hospital admissions would provide meaningful comparison ^13^. For analysis within 2020, the reference period was Period 1 which was prior to the beginning of the first COVID-19 wave. To compare population rates of healthcare utilisation between pre- and post-COVID-19 time periods, two reference periods were used; the historic reference periods and Period 1 2020. The results reported in this paper primarily focus on the comparison within 2020 using Period 1 2020 as a reference period.

**Table 1.** Rationale for study time periods.

Time Periods	Week	Date	Rationale for Definition of the Time Period
Period 1	1–8	06/01/2020–01/03/2020	Prior to the first wave of COVID-19
Period 2	9–12	02/03/2020–29/03/2020	Some restrictions in place but prior to advice being issued to stay at home
Period 3	13–19	30/03/2020–17/05/2020	Population level public health restrictions where all were advised to stay at home
Period 4	20–26	18/05/2020–05/07/2020	Phase 1, 2 and beginning of Phase 3 of the easing of restrictions

***Outcomes.*** The outcomes were presentation to and admission from ED as recorded on PET and an acute hospital admission of any type for a non-COVID-19 illness. A non-COVID-19 hospital admission was defined as a hospital discharge (including death) recorded on HIPE where the diagnosis was a non-COVID-19 illness. Patients recorded with an ICD-10 diagnostic code for COVID-19 (U071 OR U072 OR B342 OR B972) were excluded for this purpose. The occurrence and characteristics of the outcomes were compared between exposure and reference periods. In order to describe stratified rates of each outcome and the characteristics of the population who experienced outcomes for the exposure and reference periods, relevant variables were included from the PET and HIPE datasets ( Table 2). To explore trends further for selected clinical conditions to inform and aid recovery planning, ‘tracer diagnoses’ were chosen from within HIPE using defined ICD-10 codes ( Table 3). These conditions were chosen following a review of the literature and from discussions with the HSE Lead for Integrated Care, for the Acute Hospitals and for Mental Health all of whom were providing frontline clinical care. The purpose of selecting the ‘tracer diagnoses’ was to explore healthcare utilisation trends in key clinical areas where changes in healthcare utilisation had been observed. The rationale for the selection of the ‘tracer diagnoses’ is outlined in Table 3.

**Table 2.** Variables describing population characteristics.

PET Dataset	HIPE Dataset
Date of Attendance	Date of Admission
Gender	Gender
Age	Age
Discharge Destination	Principal Diagnostic Group: Clinical Classification System-Irish Modification (CCS-IM)
Mode of Arrival	Admission Source
Mode of Referral	Discharge Destination
Triage Status	Discharge Outcome: Dead or Alive
	Admission Type
	Charlson co-morbidity index (CCI)

**Table 3.** Tracer diagnoses.

Diagnosis	ICD-10 Codes	Rationale for Inclusion in Study
Stroke TIA	I60.9, I61.9, I62.9, I63.0-I63.9, I64 G45.9	Evidence internationally within the literature of reduced and delayed stroke/TIA presentations and increases in morbidity and mortality ^3, 18– 20^
STEMI NSTEMI	STEMI I21.1, I21.2, I21.3 NSTEMI I21.4 Acute MI unspecified I21.9	Evidence internationally within the literature of reduced and delayed presentations with STEMI/NSTEMI and increases in morbidity and mortality ^4, 21– 23^
Self-harm	X60-X84	Some evidence nationally within the literature of an initial reduction in presentations with self-harm followed by a rebound increase with increasing severity of presentations ^24^
Acute alcohol related presentations	F10.0-F10.9 Y90.0-Y91.0 K70.1 (acute alcoholic hepatitis) K85.2 (acute alcoholic pancreatitis) K29.2 (alcoholic gastritis)	There is limited evidence of the impact of population level restrictions and the COVID-19 pandemic on alcohol related presentations. There is evidence that presentations with self-harm had higher rates of associated substance misuse ^24^
Injury	S00-S99 T00-T31	Evidence that presentations due to injuries reduced during the population level restrictions due to the COVID-19 pandemic ^25, 26^
Road Traffic Accidents (RTAs)	V01-04, V06, V09-V79, V87, V89, V99	It would be expected that admissions due to RTAs would decrease during population level restrictions

### Data analysis

Using Census 2016 data as the denominator ^17^, overall, age-specific and gender-specific population rates for each outcome were calculated with 95% Confidence Intervals (CI) for weekly counts across the 26-week study period and total and average weekly counts across the defined sub-periods. Rate differences with 95% CIs, and rate ratios with 95% CIs were used to compare the occurrence of the outcome across exposure and reference periods. A chi-squared test was used to test the hypothesis that there was no difference between the proportion of the population who experienced an outcome across exposure and reference periods. The characteristics of those who experienced an outcome were compared between exposure period and reference periods using a chi-squared test to investigate the null hypothesis that there was no difference in the characteristics across exposure and reference periods. Using PET data, the effect of patient-level characteristics, including time period, were compared for association with the likelihood of admission from ED using a binary logistic regression model. Adjusted Odds Ratios (AOR) were calculated to measure the independent likelihood of admission from ED for a specific level of a characteristic relative to the reference level within the model. The purpose of the regression analysis was to assess if presentation to ED within the specific study time periods was associated with an increased likelihood of admission from ED as admission following ED presentation is an indicator of acuity ^27^. The multiple logistic regression analysis could only be conducted using PET data as HIPE data are aggregate data and therefore regression analysis was not suitable. Within HIPE, initial data analysis was for all admission types, which was followed by further sub-group analysis of rates of elective and emergency non-COVID-19 hospital admissions by diagnostic group (CCS-IM) ^28^. All statistical analysis was carried out using Microsoft Excel, SPSS version 26.0, Stata 15 (Stata Corporation) and Open-Source Epidemiologic Statistics for Public Health version 3.01. Level of significance for all group differences in this study was set at 5% (p-value <0.05).

### Reporting guideline

The Reporting of Studies Conducted using Observational Routinely-collected Data (RECORD) guideline extended from the Strengthening the Reporting of Observational Studies in Epidemiology (STROBE) statement on reporting guidelines was used for this study.

### Ethical approval

Ethical review was not required for this study as the research is secondary analysis of anonymised data sets. The data used in the study are controlled by the HSE in Ireland. The study authors (LM and PK) conducted data processing for the study at the HSE National Health Intelligence Unit to inform the statutory function of the HSE in Ireland to improve, promote and protect the health and welfare of the public health ^29^. HIPE data are anonymised for users and usual practices regarding statistical disclosure control were applied.

## Results

This paper primarily focuses on the results of the comparison between Periods 2–4 2020 and Period 1 2020. It also presents key comparisons using historic reference periods. The results of the internal comparison were reviewed against the historic reference period for both PET (2019) and HIPE (2017–2019) datasets and the results and public health implications are similar. Using both reference periods demonstrated that the overall trends in healthcare utilisation were similar despite seasonal differences for comparisons made within 2020.

### Overall trends - total population, gender and age group

**Figure 1.**
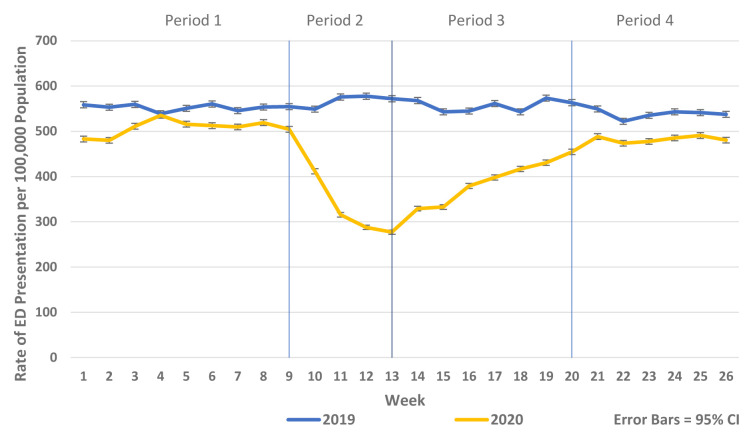
Weekly rate of ED presentation per 100,000 population week 1–26 2019 vs. 2020.

**Figure 2.**
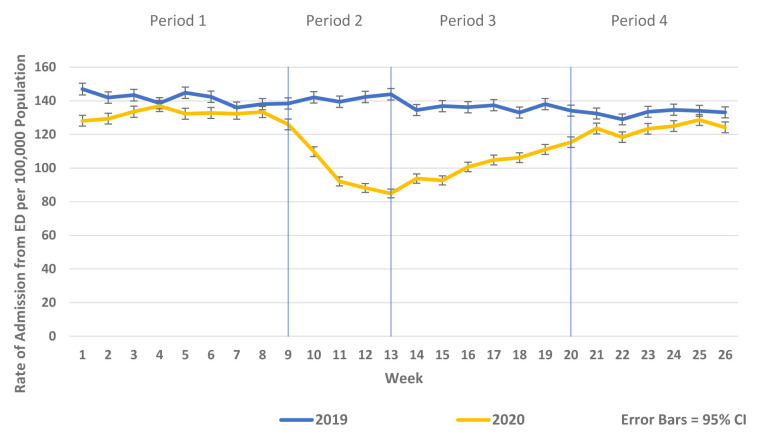
Weekly rate of admission from ED per 100,000 population week 1–26 2019 vs. 2020.

**Figure 3.**
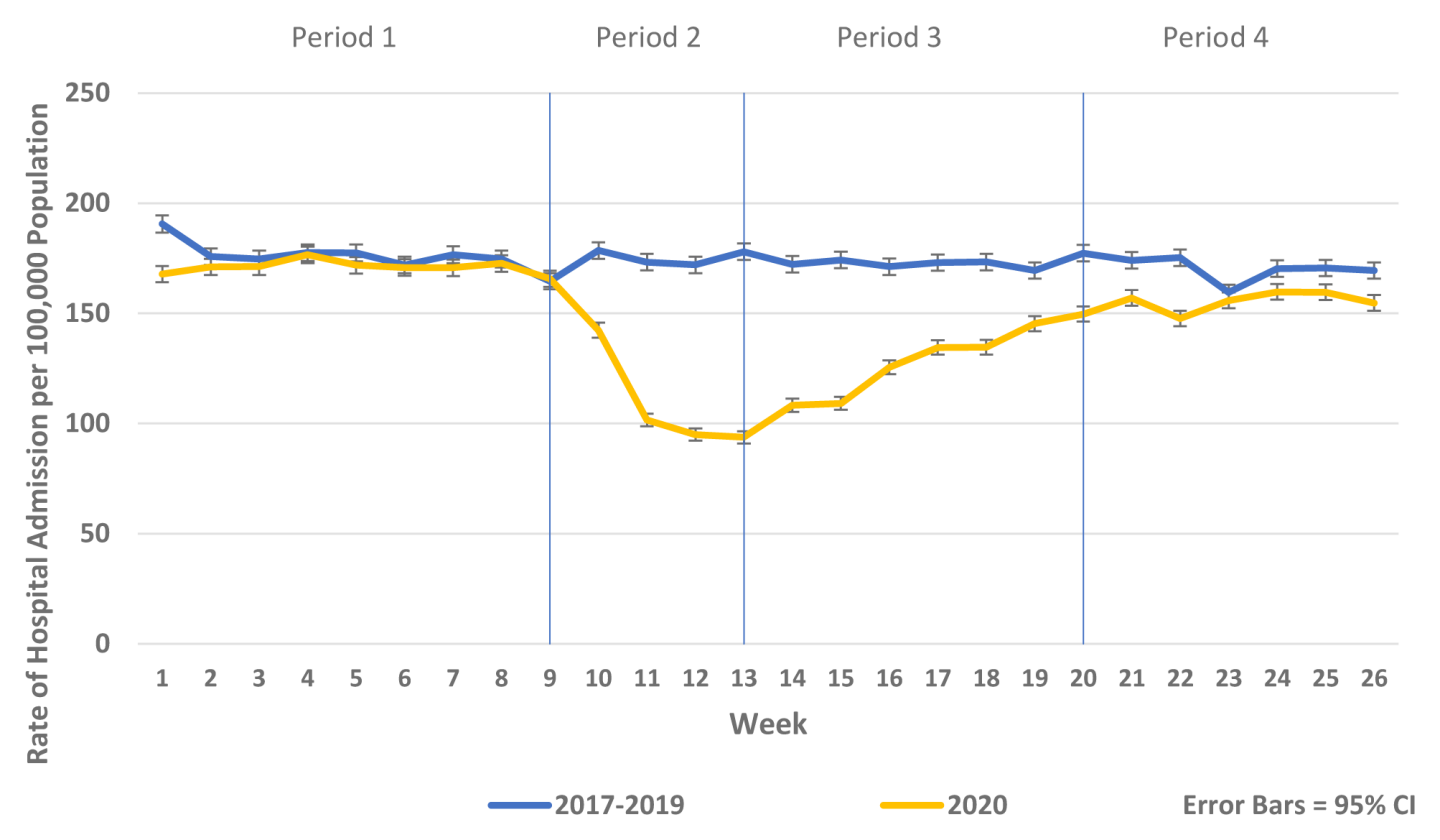
Weekly rate of non-COVID-19 emergency admission per 100,000 population 2017–19 vs. 2020.

**Figure 4.**
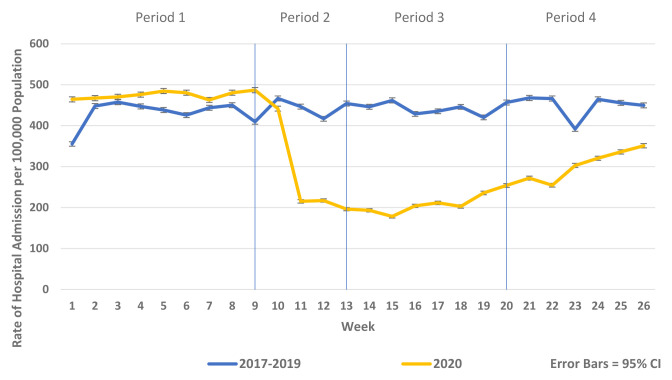
Weekly rate of non-COVID-19 elective admission per 100,000 population 2017–2019 vs 2020.

**Figure 5.**
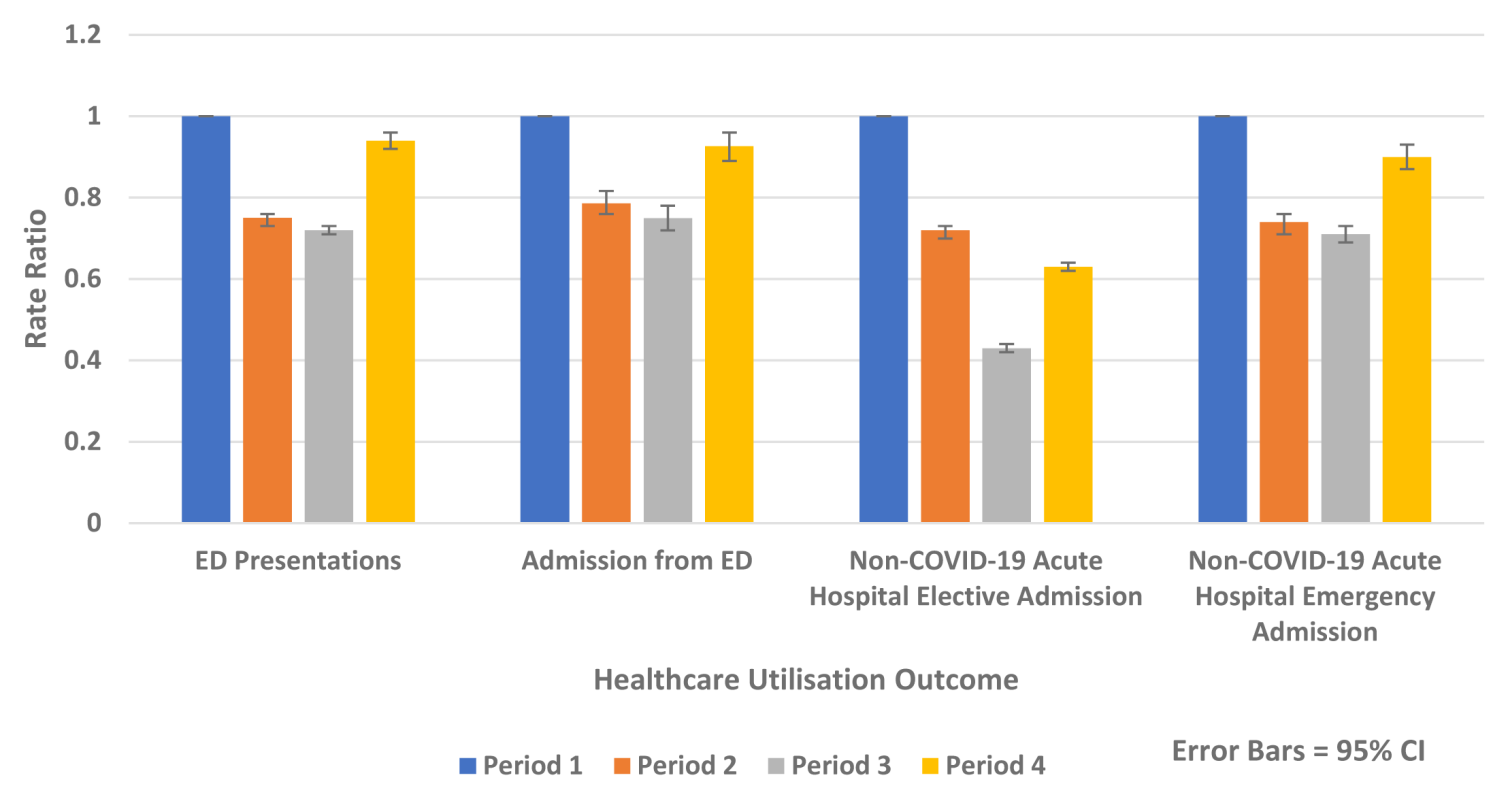
Rate ratios of average weekly ED presentation, admission from ED and non-COVID-19 acute hospital elective and emergency admission 2020.

There was a substantial reduction in population rates of ED presentation and admission from ED in Periods 2–4 2020 compared to the historic reference period in 2019 ( Figure 1 and Figure 2). Similarly, there were reductions in non-COVID-19 admissions of all types compared to historic reference periods from 2017–2019. As the reductions in non-COVID-19 hospital admissions were predominantly for elective and emergency admissions and as there were notable differences in the patterns and trends observed between elective and emergency admissions, these are presented separately in Figure 3– Figure 5 and Figure 7. Trends in elective and emergency non COVID-19 hospital admission are shown in Figure 3 and Figure 4. Figure 5 outlines the results of the internal comparison within 2020 and shows reductions in Periods 2–4 2020 compared to Period 1 2020. Figure 8 shows reductions in non-COVID-19 hospital admissions for all admission types.

The greatest absolute and relative rate reductions were seen in Period 3 2020 compared to Period 1 2020 for the following:

-   ED presentation (Rate Difference -142.1 per 100,000 population, 95% CI, -150.4, -133.6, p-value <0.0001 and Rate Ratio 0.72, 95% CI 0.71, 0.73, p-value <0.0001)

-   Admission from ED (Rate Difference -33.2 per 100,00 population, 95% CI, -37.5, -28.9, p-value <0.0001, Rate Ratio 0.75, 95% CI 0.72, 0.78, p-value <0.0001)

-   Overall non-COVID-19 acute hospital admission (Rate Difference -329.5 per 100,00 population, 95% CI, -338.8, -320.2, p-value <0.0001 and Rate Ratio 0.53, 95% CI 0.52, 0.54, p-value <0.0001)

-   Non-COVID-19 emergency hospital admission (Rate Difference -50.0 per 100,00 population, 95% CI, -54.9, -45.2, p-value <0.0001 and Rate Ratio 0.71, 95% CI 0.69, 0.73, p-value <0.0001)

-   Non-COVID-19 elective hospital admission (Rate Difference -270.2 per 100,00 population, 95% CI, -277.6, -262.9, p-value <0.0001 and Rate Ratio, 0.43 95% CI, 0.42, 0.44, p-value <0.0001)

**Figure 6.**
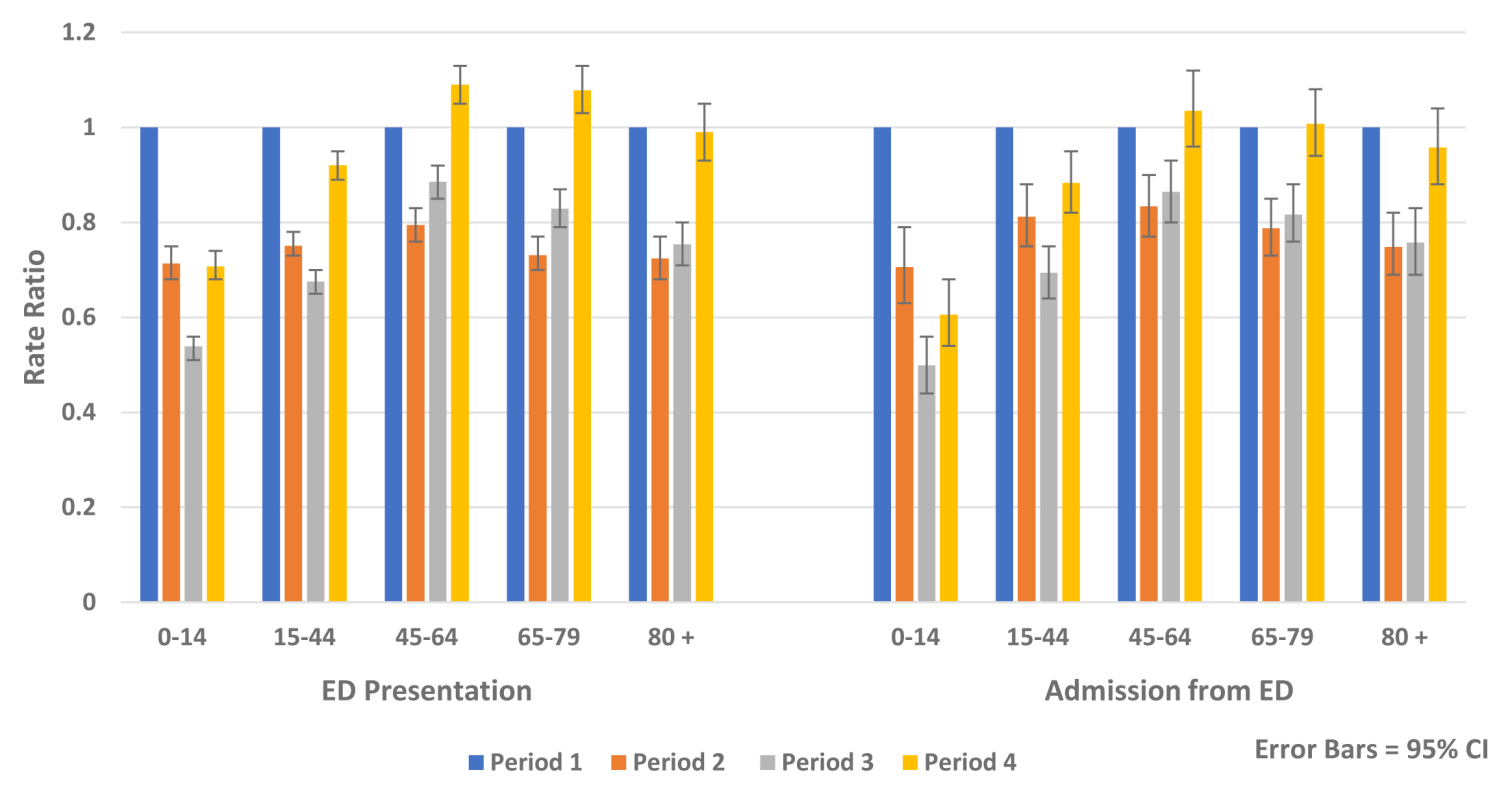
Rate ratio of average weekly ED presentation and admission from ED and non-COVID-19 hospital admission by age 2020.

**Figure 7.**
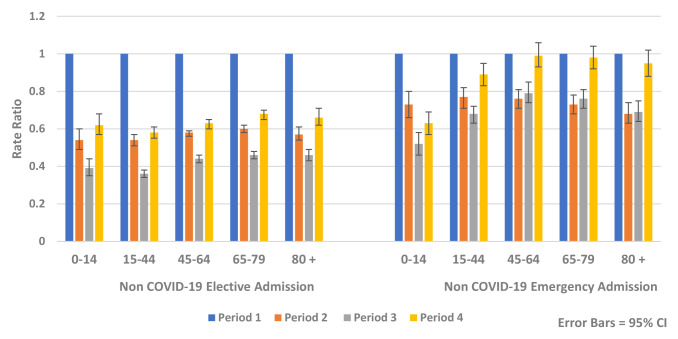
Rate ratio of average weekly non-COVID-19 elective and emergency admission by age 2020.

Similar reductions were observed for both genders and across all age groups. The greatest relative rate reductions were in younger age groups (<45 years) while the greatest absolute rate reductions were seen in older age groups, particularly those aged over 80 years ( Figure 6 and Figure 7).

**Figure 8.**
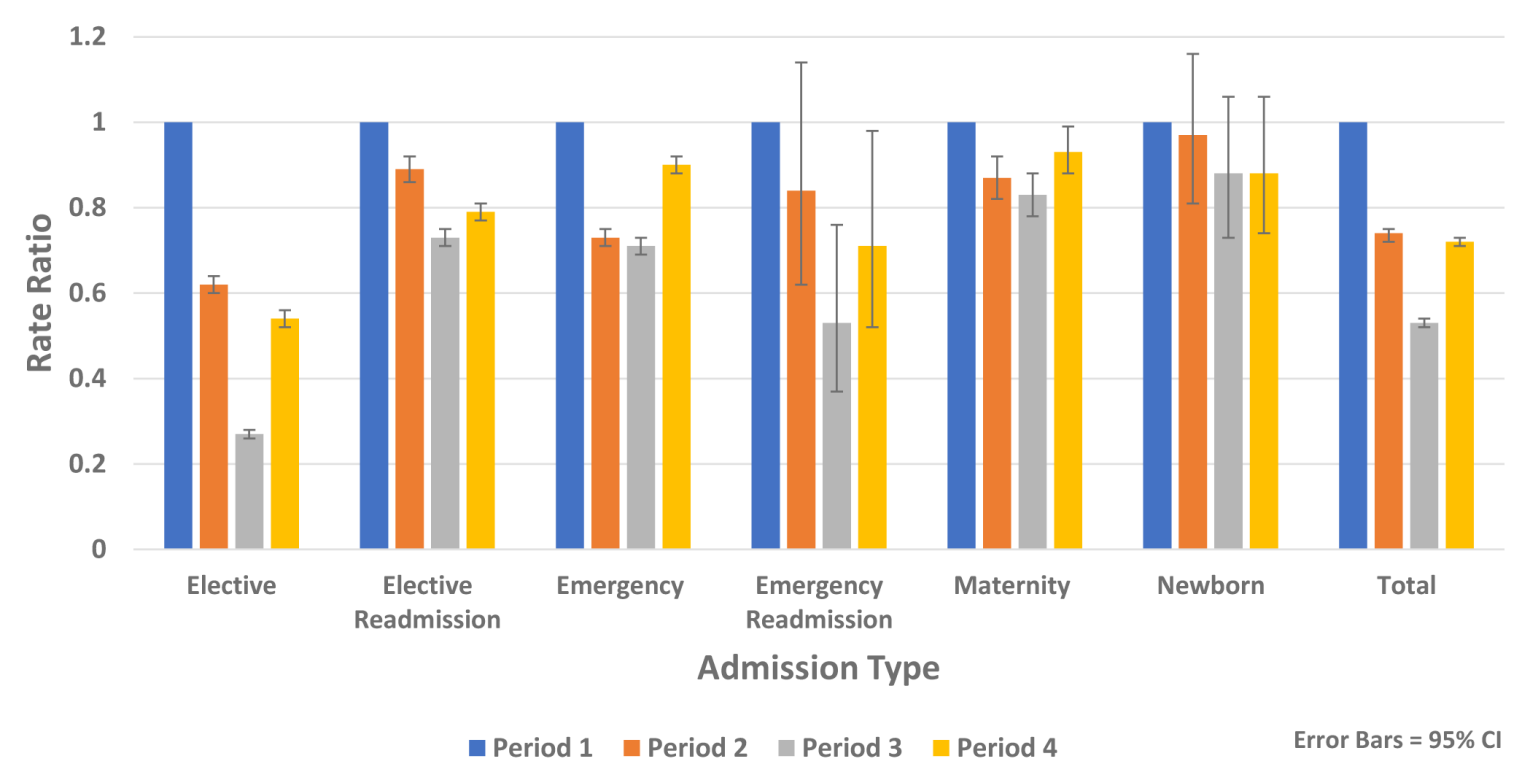
Rate ratios of average weekly non-COVID-19 admission by admission type 2020.

Within Periods 2–4 there were 81,712 fewer ED presentations (-18.8%), 19,692 fewer admissions from ED (-17.4%) and 210,357 fewer non-COVID-19 hospital admissions (-35.0%) than what would have been expected based on Period 1 2020. This included 173,688 fewer elective care admissions (-42.8%) and 30,899 fewer non-COVID-19 emergency care admissions (21.0%) (Supplementary Tables A1–A3).

### Trends in emergency department presentations and admissions

Analysis of ED activity indicated that a greater proportion of those presenting to ED in Periods 2–4 2020 were admitted and a greater proportion of both presentations and admissions were from older age groups (p-value <0.0001), had arrived by ambulance (p-value <0.0001) and were admitted (p-value <0.0001) compared to Period 1 2020 ( Table 4 and Table 5). Factors associated with conversion to hospital admission following presentation to ED were examined for their independent association through a logistic regression model ( Table 6). Being in a higher triage category (AOR 8.88, 95% CI 8.64, 9.13, p-value <0.0001), followed by older age (AOR 5.00, 95% CI 4.84, 5.17, p-value <0.0001) were the greatest predictors of hospital admission from ED. Independent of other factors included in the model, there was an increased likelihood of being admitted to hospital following ED presentation in Periods 2–4 compared to Period 1, which was most marked in Period 3 (AOR, 1.10, 95% CI, 1.07, 1.12, p-value <0.0001). In Period 4, ED presentations and admissions from ED returned to pre-COVID-19 levels for those aged over 45 years but remained reduced for those aged below 45 years ( Figure 6).

**Table 4.** Comparison of the characteristics of the population that presented to ED period 2–4 2020 vs period 1 2020.

ED Presentation	Period 1 (Reference) Week 1–8		Period 2 Week 9–12		Period 3 Week 13–19		Period 4 Week 20–26	
Mode of Arrival	N	%	N	%	N	%	N	%
Total Weekly Average	22,125.2	100.0	16,669.3	100.0	15,861.9	100.0	20,701.3	100.0
Ambulance/ Helicopter	5,031.9	22.7	4,450.5	26.7	3,906.6	24.6	4,496.4	21.7
Other	17,093.3	77.3	12,218.8	73.3	11,955.3	75.4	16,204.9	78.3
χ2 (p-value)	-	-	-	417.65 (<0.0001)	-	135.11 (<0.0001)	-	47.94 (<0.0001)
Mode of Referral	N	%	N	%	N	%	N	%
Total Weekly Average	23,583.1	100.0	17,597.6	100.0	17,042.7	100.0	22,279.5	100.0
GP/GP OOH	8,416.0	35.7	5,434.0	30.9	5,301.7	31.1	7,109.7	31.9
Self-Referral	12,636.8	53.6	10,243.8	58.2	10,047.7	59.0	12,999.7	58.3
Other	2,530.3	10.7	1,919.8	10.9	1,693.3	9.9	2,170.1	9.8
χ2 (p-value)	-	-	-	546.28 (<0.0001)	-	874.77 (<0.0001)	-	786.95 (<0.0001)
Triage Category	N	%	N	%	N	%	N	%
Total Weekly Average	18,754.4	100.0	14,115.5	100.0	13,692.4	100.0	17,974.9	100.0
Immediate/V urgent	4,225.3	22.5	3,281.5	23.3	2,917.7	21.3	3,624.1	20.2
Urgent	9,637.1	51.4	7,186.0	50.9	7,232.1	52.8	9,302.9	51.7
Standard/Non-Urgent	4,892.0	26.1	3,648.0	25.8	3,542.6	25.9	5,047.9	28.1
χ2 (p-value)	-	-	-	12.05 (<0.0001)	-	63.62 (<0.0001)	-	281.80 (<0.0001)
Discharge Destination	N	%	N	%	N	%	N	%
Total Weekly Average	23,534.0	100.0	17,573.5	100.0	16,208.4	100.0	21,224.1	100.0
Admitted	6,303.0	26.8	4,953.5	28.2	4,722.0	29.1	5,842.0	27.5
Not Admitted	17,231.0	73.2	12,620.0	71.8	11,486.4	70.9	15,382.1	72.5
χ2 (p-value)	-	-	-	51.05 (<0.0001)	-	195.44 (<0.0001)	-	23.18 (<0.0001)
Age Group	N	%	N	%	N	%	N	%
Total Weekly Average	24,196.3	100.0	18,077.4	100.0	17,434.4	100.0	22,783.4	100.0
Age 0–14	4,895.1	20.2	3,493.5	19.3	2,637.9	15.1	3,463.4	15.2
Age 15–44	8,659.8	35.8	6,503.0	36.0	5,849.1	33.5	7,971.0	35.0
Age 45–64	4,971.6	20.5	3,949.8	21.8	4,404.1	25.3	5,420.3	23.8
Age 65–79	3,599.0	14.9	2,631.8	14.6	2,982.4	17.1	3,878.7	17.0
Age 80+	2,070.8	8.6	1,499.3	8.3	1,560.9	9.0	2,050.0	9.0
χ2	-	-	-	72.70 (<0.0001)	-	2,168.12 (<0.0001)	-	193.80 (<0.0001)
Gender	N	%	N	%	N	%	N	%
Total Weekly Average	24,203.5	100.0	18,084.8	100.0	17,440.0	100.0	22,791.1	100.0
Males	12,195.9	50.4	9,305.0	51.5	8,763.4	50.2	11,504.8	50.4
Females	12,007.6	49.6	8,779.8	48.5	8,676.6	49.8	11,286.3	49.6
χ2 (p-value)	-	-	-	23.82 (<0.0001)	-	0.59 (0.444)	-	0.29 (0.592)

**Table 5.** Comparison of the characteristics of the population admitted from ED period 2–4 2020 vs period 1 2020.

Admission from ED	Period 1 (Reference) Week 1–8		Period 2 Week 9–12		Period 3 Week 13–19		Period 4 Week 20–26	
Mode of Arrival	N	%	N	%	N	%	N	%
Total Weekly Average	5,927.0	100.0	4,662.6	100.0	4,364.4	100.0	5,402.6	100.0
Ambulance/ Helicopter	2,452.1	41.4	2,222.3	47.7	2,050.7	47.0	2,263.0	41.9
Other	3,474.9	58.6	2,440.3	52.3	2,313.7	53.0	3,139.6	58.1
χ ^2^ (p-value)	-	-	-	215.89 (<0.0001)	-	238.24 (<0.0001)	-	2.31 (0.129)
Mode of Referral	N	%	N	%	N	%	N	%
Total Weekly Average	6,137.7	100.0	4,821.6	100.0	4,604.2	100.0	5,726.4	100.0
GP/GP OOH Referral	2,309.4	37.6	1,498.8	31.1	1,392.0	30.2	1,908.3	33.3
Self-Referral	3,029.9	49.4	2,684.5	55.7	2,608.6	56.7	3,084.0	53.9
Other	798.4	13.0	638.3	13.2	603.6	13.1	734.1	12.8
χ ^2^ (p-value)	-	-	-	274.04 (<0.0001)	-	504.61 (<0.0001)	-	201.63 (<0.0001)
Triage Category	N	%	N	%	N	%	N	%
Total Weekly Average	4,938.5	100.0	3,860.0	100.0	3,665.5	100.0	4,551.5	100.0
Immediate/V urgent	2,122.4	43.0	1,696.0	43.9	1,510.4	41.2	1,794.9	39.4
Urgent N	2,403.8	48.7	1,844.5	47.8	1,865.0	50.9	2,364.3	52.0
Standard/Non-Urgent	412.3	8.3	319.5	8.3	290.1	7.9	392.3	8.6
χ ^2^ (p-value)	-	-	-	4.25 (0.119)	-	30.36 (<0.0001)	-	92.63 (<0.0001)
Age Group	N	%	N	%	N	%	N	%
Total Weekly Average	6,303.0	100.0	4,953.2	100.0	4,721.8	100.0	5,841.7	100.0
Age 0–14	778.8	12.4	549.8	11.1	388.4	8.2	471.9	8.1
Age 15–44	1,416.1	22.5	1,149.8	23.2	982.1	20.8	1,251.0	21.4
Age 45–64	1,370.8	21.7	1,142.8	23.1	1,185.1	25.1	1,418.4	24.3
Age 65–79	1,575.9	25.0	1,241.8	25.1	1,286.3	27.3	1,588.3	27.2
Age 80+	1,161.4	18.4	869.0	17.5	879.9	18.6	1,112.1	19.0
χ ^2^ (p-value)	-	-	-	39.56 (<0.0001)	-	479.30 (<0.0001)	-	516.61 (<0.0001)
Gender	N	%	N	%	N	%	N	%
Total Weekly Average	6,302.9	100.0	4,953.6	100.0	4,722.0	100.0	5,842.0	100.0
Males	3,155.0	50.1	2,561.8	51.7	2,440.1	51.7	2,956.7	50.6
Females	3,147.9	49.9	2,391.8	48.3	2,281.9	48.3	2,885.3	49.4
χ ^2^ (p-value)	-	-	-	15.67 (<0.0001)	-	20.95 (<0.0001)	-	2.78 (0.095)

**Table 6.** Predictors of admission from ED in 2020.

Variables in Logistic Regression Model	Total ED Presentations		Admitted		Not Admitted		Adjusted OR	95% CI lower, upper	p-value
Time Period	N	%	N	%	N	%			
Period 1 2020 *	188,272	100.0	50,424	26.8	137,848	73.2	1.00	-	-
Period 2 2020	70,294	100.0	19,814	28.2	50,480	71.8	1.04	1.01, 1.07	0.005
Period 3 2020	113,459	100.0	33,054	29.1	80,405	70.9	1.10	1.07, 1.12	<0.0001
Period 4 2020	148,569	100.0	40,894	27.5	107,675	72.5	1.06	1.04,1.08	<0.0001
**Age Category**									
Age 0–14 *	94,383	100.0	14,451	15.3	79,932	84.7	1.00	-	-
Age 15–44	181,178	100.0	31,560	17.4	149,618	82.6	1.08	1.05, 1.11	<0.0001
Age 45–64	117,068	100.0	33,762	28.8	83,306	71.2	1.89	1.84, 1.94	<0.0001
Age 65–79	82,354	100.0	37,696	45.8	44,658	54.2	3.57	3.47, 3.67	<0.0001
Age 80+	45,418	100.0	26,711	58.8	18,707	41.2	5.00	4.84, 5.17	<0.0001
**Triage Category**									
Standard/Non-Urgent *	110,092	100.0	9,353	8.5	100,739	91.5	1.00	-	-
Immediate/Very Urgent	89,739	100.0	46,900	52.3	42,839	47.7	8.88	8.64, 9.13	<0.0001
Urgent	209,769	100.0	56,213	26.8	153,556	73.2	3.19	3.11, 3.28	<0.0001
**Mode of Referral**									
Self-Referral *	289,266	100.0	74,825	25.9	214,441	74.1	1.00	-	-
GP/GP OOH Referral	165,372	100.0	47,572	28.8	117,800	71.2	1.51	1.48,1.54	<0.0001
Other Mode of Referral	53,458	100.0	18,304	34.2	35,154	65.8	1.37	1.33,1.41	<0.0001
**Mode of Arrival**									
Mode of Arrival Other Mode of Arrival *	363,776	100.0	75,733	20.8	288,043	79.2	1.00	-	-
Arrival by Ambulance/ Helicopter	111,487	100.0	58,702	52.7	52,785	47.3	2.16	2.12, 2.21	<0.0001
**Gender**									
Male *	263,231	100.0	73,265	27.8	189,966	72.2	1.00	-	-
Female	257,348	100.0	70,920	27.6	186,428	72.4	1.00	0.99, 1.02	0.805
**Total N 375,822**	**Nagelkerke R ^2^ 28.6%**			**χ ^2^ 83184.65 (p-value** **<0.0001)**			**Degrees of Freedom 13**		

*Reference Category

### Trends in non-COVID-19 hospital admissions

Analysis of non-COVID-19 hospital admissions using HIPE data found reductions across all diagnostic groups and all admission types including elective, emergency, maternity and newborn admissions ( Figure 8).

Trends in elective and emergency admissions for selected diagnostic groups are shown in Table 7. Comparing elective admissions in Periods 2–4 2020 to what would have been expected based on Period 1 2020, there were particularly large reductions in cancer (36,120 fewer episodes of admission, -33.8%), gastroenterology (26,895 fewer episodes of admission, -56.1%), dermatology (12,180 fewer episodes of admission, -66.8%), respiratory (8,021 fewer episodes of admission, -65.8%) and cardiovascular (6,637 fewer episodes of admission, -58.5%) admissions. Further analysis of emergency admissions with specific selected ‘tracer diagnoses’ showed reductions in admissions with stroke and transient ischaemic attack (TIA) (411 fewer episodes of admission, -12.1%) and acute myocardial infarction (AMI) (395 fewer episodes of admission, -14.7%) in Periods 2–4 2020 compared to expected based on Period 1 2020. There were also reductions in emergency admission with injury (2,059 fewer episodes of admission, -21.4%) and post-road traffic accident (RTA) (182 fewer episodes of admission, -24.4%) in Periods 2 and 3 2020 compared to expected based on Period 1 ( Table 8).

**Table 7.** Weekly average non-COVID-19 elective and emergency admissions by CCS-IM 2020.

	Period 1 (Ref) Week 1–8		Period 2 Week 9–12		Period 3 Week 13–19		Period 4 Week 20–26	
Total Admission	Elective	Emergency	Elective	Emergency	Elective	Emergency	Elective	Emergency
Weekly Average Count	22,534.6	8,171.5	16,193.8	6,007.3	9,665.3	5,789.9	14,214.6	7,375.6
Weekly Average Rate *	473.2	171.6	340.1	126.2	203.0	121.6	298.5	154.9
Count Difference	-	-	-6,340.8	-2,164.2	-12,869.3	-2,381.6	-8,320.0	-795.9
Rate Difference (95% CI)	-	-	-133.1 (-141.2, -125.1)	-45.4 (-50.4, -40.6)	-270.2 (-277.6, -262.9)	-50.0 (-54.9, -45.2)	-174.7 (-182.6, -166.8)	-16.7 (-21.8, 11.6)
Rate Ratio (95% CI)	-	-	0.72 (0.70, 0.73)	0.74 (0.71, 0.76)	0.43 (0.42, 0.44)	0.71 (0.69, 0.73)	0.63 (0.62, 0.64)	0.90 (0.87, 0.93)
χ ^2^ (p-value)	-	-	1,042 (<0.0001)	330.80 (<0.0001)	5,161 (<0.0001)	406.90 (<0.0001)	1,891 (<0.0001)	40.81 (<0.0001)
Cancer Admission	Period 1		Period 2		Period 3		Period 4	
	Elective	Emergency	Elective	Emergency	Elective	Emergency	Elective	Emergency
Weekly Average Count	5,931.4	268.4	4,691.3	193.3	3,226.4	209.3	4,185.1	249.6
Weekly Average Rate	124.6	5.6	98.5	4.1	67.8	4.4	87.9	5.2
Count Difference	-	-	-1,240.1	-75.1	-2,705.0	-59.1	-1,746.3	-18.8
Rate Difference (95% CI)	-	-	-26.1 (-30.3, -21.8)	-1.5 (-2.5, -0.7)	-56.8 (-60.7, -52.9)	-1.2 (-2.1, -0.3)	-36.7 (-40.8, -32.5)	-0.4 (-1.3, 0.5)
Rate Ratio (95% CI)	-	-	0.79 (0.76, 0.82)	0.72 (0.59, 0.87)	0.54 (0.52, 0.57)	0.78 (0.65, 0.93)	0.71 (0.68, 0.73)	0.93 (0.78, 1.11)
χ ^2^ (p-value)	-	-	144.90 (<0.0001)	12.23 (0.001)	799.70 (<0.0001)	7.31 (0.007)	301.70 (<0.0001)	0.68 (0.409)
Cardiovascular Admission	Period 1		Period 2		Period 3		Period 4	
	Elective	Emergency	Elective	Emergency	Elective	Emergency	Elective	Emergency
Weekly Average Count	630.3	1,416.3	384.5	980.3	155.0	1,145.1	297.9	1,431.3
Weekly Average Rate	13.2	29.7	8.1	20.6	3.3	24.0	6.3	30.1
Count Difference	-	-	-245.8	-436.0	-475.3	-271.2	-332.4	15.0
Rate Difference (95% CI)	-	-	-5.1 (-6.5, -3.9)	-9.1 (-11.2, -7.1)	-9.9 (-11.1, -8.8)	-5.7 (-7.8, -3.6)	-6.9 (-8.2, -5.7)	0.4 (-1.9, 2.5)
Rate Ratio (95% CI)	-	-	0.61 (0.54, 0.69)	0.69 (0.64, 0.75)	0.25 (0.21, 0.29)	0.81 (0.75, 0.87)	0.47 (0.41, 0.54)	1.01 (0.94, 1.09)
χ ^2^ (p-value)	-	-	59.52 (<0.0001)	79.34 (<0.0001)	287.70 (<0.0001)	28.70 (<0.0001)	119.10 (<0.0001)	0.08 (0.778)
Gastroenterology Admission	Period 1		Period 2		Period 3		Period 4	
	Elective	Emergency	Elective	Emergency	Elective	Emergency	Elective	Emergency
Weekly Average Count	2,662.9	940.8	1,623.0	694.0	644.3	743.3	1,433.6	928.4
Weekly Average Rate	55.9	19.8	34.1	14.6	13.5	15.6	30.1	19.5
Count Difference	-	-	-1,039.9	-246.8	-2,018.6	-197.5	-1,229.3	-12.4
Rate Difference (95% CI)	-	-	-21.8 (-24.5, -19.1)	-5.2 (-6.9, -3.5)	-42.4 (-44.8, -40.0)	-4.2 (-5.8, -2.5)	-25.8 (-28.4, -23.2)	-0.3 (-0.2, 1.5)
Rate Ratio (95% CI)	-	-	0.61 (0.57, 0.65)	0.74 (0.67, 0.81)	0.24 (0.22, 0.26)	0.79 (0.72, 0.87)	0.54 (0.50, 0.57)	0.99 (0.90, 1.08)
χ ^2^ (p-value)	-	-	252.41 (<0.0001)	37.25 (<0.0001)	1,233 (<0.0001)	23.16 (<0.0001)	369.12 (<0.0001)	0.08 (0.776)
Mental Health Admission	Period 1		Period 2		Period 3		Period 4	
	Elective	Emergency	Elective	Emergency	Elective	Emergency	Elective	Emergency
Weekly Average Count	16.5	136.9	10.8	94.3	4.0	130.4	6.6	175.6
Weekly Average Rate	0.3	2.9	0.2	2.0	0.1	2.7	0.1	3.7
Count Difference	-	-	-5.7	-42.6	-12.5	-6.5	-9.9	38.7
Rate Difference (95% CI)	-	-	-0.1 (-0.3, -0.1)	-0.9 (-1.5, -0.3)	-0.2 (-0.5, -0.1)	-0.2 (-0.1, 0.5)	-0.2 (-0.4, -0.01)	0.8 (0.1, 1.5)
Rate Ratio (95% CI)	-	-	0.65 (0.30, 1.41)	0.69 (0.53, 0.89)	0.24 (0.08, 0.72)	0.95 (0.75, 1.21)	0.39 (0.16, 0.98)	1.28 (1.03, 1.60)
χ ^2^ (p-value)	-	-	1.21 (0.2718)	7.86 (0.005)	7.62 (0.0058)	0.16 (0.690)	4.27 (0.0387)	4.79 (0.028)
Respiratory Admission	Period 1		Period 2		Period 3		Period 4	
	Elective	Emergency	Elective	Emergency	Elective	Emergency	Elective	Emergency
Weekly Average Count	677.6	1,549.8	426.3	1,261.3	125.0	773.9	228.0	769.9
Weekly Average Rate	14.2	32.5	9.0	26.5	2.6	16.3	4.8	16.2
Count Difference	-	-	-251.3	-288.5	-552.6	-775.9	-449.6	-779.9
Rate Difference (95% CI)	-	-	-5.2 (-6.7, -3.9)	-6.0 (-8.2, -3.9)	-11.6 (-12.8, -10.4)	-16.2 (-18.3, -14.3)	-9.4 (-10.7, -8.2)	-16.3 (-18.4, -14.4)
Rate Ratio (95% CI)	-	-	0.63 (0.56, 0.71)	0.81 (0.76, 0.88)	0.18 (0.15, 0.22)	0.49 (0.46, 0.54)	0.34 (0.28, 0.39)	0.49 (0.46, 0.54)
χ ^2^ (p-value)	-	-	57.25 (<0.0001)	29.62 (<0.0001)	380.51 (<0.0001)	259.10 (<0.0001)	223.31 (<0.0001)	262.30 (<0.0001)
Infection Admission	Period 1		Period 2		Period 3		Period 4	
	Elective	Emergency	Elective	Emergency	Elective	Emergency	Elective	Emergency
Weekly Average Count	229.9	145.3	118.8	119.8	16.9	91.4	48.6	104.9
Weekly Average Rate	4.8	3.1	2.5	2.5	0.4	1.9	1.0	2.2
Count Difference	-	-	-111.1	-25.5	-213.0	-53.9	-181.3	-40.4
Rate Difference	-	-	-2.3 (-3.1, -1.6)	-0.6 (-1.2, 0.1)	-4.4 (-5.1, -3.8)	-1.2 (-1.8, -0.5)	-3.8 (-4.5, -3.1)	-0.9 (-1.5, -0.2)
Rate Difference (95% CI)	-	-	0.52 (0.41, 0.64)	0.82 (0.65, 1.05)	0.07 (0.04, 0.12)	0.63 (0.48, 0.82)	0.21 (0.15. 0.29)	0.72 (0.56. 0.93)
χ ^2^ (p-value)	-	-	35.43 (<0.0001)	2.45 (0.117)	183.90 (<0.0001)	12.24 (<0.0001)	118.10 (<0.0001)	6.52 (0.011)
Injury & Poisoning Admission	Period 1		Period 2		Period 3		Period 4	
	Elective	Emergency	Elective	Emergency	Elective	Emergency	Elective	Emergency
Weekly Average Count	245.4	1,012.3	205.0	799.5	116.1	782.1	184.3	1,043.1
Weekly Average Rate	5.2	21.3	4.3	16.8	2.4	16.4	3.9	21.9
Count Difference	-	-	-40.4	-212.8	-129.3	-230.2	-61.1	30.8
Rate Difference (95% CI)	-	-	-0.9 (-1.7, 0.03)	-4.5 (-6.2, -2.7)	-2.8 (-3.5, -1.9)	-4.9 (-6.6, -3.1)	-1.3 (-2.1, -0.4)	0.6 (-1.2, 2.5)
Rate Ratio (95% CI)	-	-	0.84 (0.69, 1.01)	0.79 (0.72, 0.87)	0.47 (0.38, 0.59)	0.77 (0.70, 0.85)	0.75 (0.62, 0.91)	1.03 (0.95, 1.12)
χ ^2^ (p-value)	-	-	3.62 (0.0571)	24.99 (<0.0001)	46.20 (<0.0001)	29.51 (<0.0001)	8.69 (0.003)	0.46 (0.496)
Dermatology Admission	Period 1		Period 2		Period 3		Period 4	
	Elective	Emergency	Elective	Emergency	Elective	Emergency	Elective	Emergency
Weekly Average Count	1,013.1	206.6	560.8	136.3	125.0	131.3	419.6	184.9
Weekly Average Rate	21.3	4.3	11.8	2.9	2.6	2.8	8.8	3.9
Count Difference	-	-	-452.3	-70.3	-888.1	-75.3	-593.5	-21.7
Rate Difference (95% CI)	-	-	-9.5 (-11.1, -7.9)	-1.4 (-2.2, -0.7)	-18.7 (-20.0, -17.3)	-1.5 (-2.3, -0.8)	-12.5 (14.0, -10.9)	-0.4 (-1.3, 0.4)
Rate Ratio (95% CI)	-	-	0.55 (0.49, 0.61)	0.66 (0.53, 0.82)	0.12 (0.10, 0.15)	0.64 (0.51, 0.79)	0.41 (0.37, 0.46)	0.89 (0.73, 1.09)
χ ^2^ (p-value)	-	-	129.91 (<0.0001)	14.45 (0.0001)	693.10 (<0.0001)	16.80 (<0.0001)	245.91 (<0.0001)	1.21 (0.272)

*All Rates per 100,000 Population

**Table 8.** Emergency admission with a tracer condition 2020.

Stroke/TIA Admission	Period 1 (Reference)	Period 2	Period 3	Period 4
Total N=4,503	Week 1–8	Week 9–12	Week 13–19	Week 20–26
Weekly Average Count	189.0	147.0	154.3	189.0
Weekly Average Rate *	4.0	3.1	3.2	4.0
Count Difference	-	-42.0	-34.7	0.0
Rate Difference (95% CI)	-	-0.9 (-1.6, -1.3)	-0.8 (-1.5, 0.03)	0.0 (-0.8, 0.8)
Rate Ratio (95% CI)	-	0.78 (0.63, 0.96)	0.82 (0.66, 1.01)	1.00 (0.82, 1.22)
χ2 (p-value)	-	5.25 (0.022)	3.51 (0.061)	0.00 (0.999)
AMI Admission	Period 1 (Reference)	Period 2	Period 3	Period 4
Total N=3,492	Week 1–8	Week 9–12	Week 13–19	Week 20–26
Weekly Average Count	149.5	116.8	124.4	136.9
Weekly Average Rate *	3.1	2.5	2.6	2.9
Count Difference	-	-32.7	-25.1	-12.6
Rate Difference (95% CI)	-	-0.6 (-1.3, -0.02)	-0.5 (-1.2, 0.2)	-0.2 (-0.9, 0.4)
Rate Ratio (95% CI)	-	0.78 (0.61, 0.99)	0.83 (0.66, 1.06)	0.92 (0.73, 1.16)
χ2 (p-value)	-	4.02 (0.045)	2.30 (0.129)	0.55 (0.457)
Alcohol Admission	Period 1 (Reference)	Period 2	Period 3	Period 4
Total N=7,150	Week 1–8	Week 9–12	Week 13–19	Week 20–26
Weekly Average Count	269.6	207.5	266.0	328.7
Weekly Average Rate *	5.7	4.4	5.6	6.9
Count Difference	-	-62.1	-3.6	59.1
Rate Difference (95% CI)	-	-1.3 (-2.2, -0.4)	-0.1 (-1.0, 0.9)	1.2 (0.2, 2.3)
Rate Ratio (95% CI)	-	0.77 (0.64, 0.92)	0.98 (0.83, 1.17)	1.22 (1.03, 1.43)
χ2 (p-value)	-	8.08 (0.005)	0.02 (0.876)	5.84 (0.016)
Self-Harm Admission	Period 1 (Reference)	Period 2	Period 3	Period 4
Total N=1,903	Week 1–8	Week 9–12	Week 13–19	Week 20–26
Weekly Average Count	64.8	74.5	65.3	90.0
Weekly Average Rate *	1.4	1.6	1.4	1.9
Count Difference	-	9.7	0.5	25.2
Rate Difference (95% CI)	-	0.2 (-0.3, 0.7)	0.0 (-0.5, 0.5)	0.5 (0.17, 1.04)
Rate Ratio (95% CI)	-	1.15 (0.82, 1.60)	1.01 (0.71, 1.42)	1.39 (1.01, 1.91)
χ2 (p-value)	-	0.68 (0.411)	0.002 (0.960)	4.10 (0.043)
RTA Admission	Period 1 (Reference)	Period 2	Period 3	Period 4
Total N=1,719	Week 1–8	Week 9–12	Week 13–19	Week 20–26
Weekly Average Count	67.8	50.0	52.0	87.6
Weekly Average Rate *	1.4	1.1	1.1	1.8
Count Difference	-	-17.8	-15.8	19.8
Rate Difference (95% CI)	-	-0.3 (-8.2, 0.1)	-0.3 (-0.8, 0.1)	0.4 (-0.1, 0.9)
Rate Ratio (95% CI)	-	0.74 (0.51, 1.06)	0.77 (0.53, 1.10)	1.29 (0.94, 1.78)
χ2 (p-value)	-	2.68 (0.102)	2.07 (0.150)	2.54 (0.111)
Injury Admission	Period 1 (Reference)	Period 2	Period 3	Period 4
Total N=21,119	Week 1–8	Week 9–12	Week 13–19	Week 20–26
Weekly Average Count	875.0	675.3	695.0	936.1
Weekly Average Rate *	18.4	14.2	14.6	19.7
Count Difference	-	-199.7	-180.0	61.1
Rate Difference (95% CI)	-	-4.2 (-5.8, -2.6)	-3.8 (5.4, -2.2)	1.3 (-0.5, 3.0)
Rate Ratio (95% CI)	-	0.77 (0.70. 0.85)	0.79 (0.72, 0.88)	1.07 (0.98, 1.17)
χ2 (p-value)	-	25.73 (<0.0001)	20.64 (<0.0001)	2.06 (0.151)

*All Rates per 100,000 Population

For all non-COVID-19 hospital admission types there was a small overall increase in in-hospital mortality in Period 3 compared to Period 1 (0.9% vs. 0.6%, p-value 0.004) and a higher proportion of patients discharged in Periods 2–4 had a Charlson co-morbidity index (CCI) ^30^ score of over 10 compared to Period 1 (19.9% vs. 13.5%, p-value <0.0001). Patients experiencing an emergency admission are generally more acutely unwell compared to other admission types. These observed differences in outcomes were no longer statistically significant when analysis was limited to emergency admissions only. In-hospital mortality for emergency admissions was 2.5% in Period 3 vs. 2.4% in Period 1 (p-value 0.888), while the proportion of those with a CCI score of over 10 was 12.2% vs. 11.7% (p-value 0.627) ( Table 9) Analysis of specific emergency tracer diagnoses also showed no difference in severity as measured with CCI and in-hospital mortality.

**Table 9.** Comparison of the characteristics of emergency non-COVID-19 admissions period 2–4 2020 vs. period 1 2020.

Emergency Admissions	Period 1 2020 (Reference)		Period 2 2020		Period 3 2020		Period 4 2020	
	Week 1–8		Week 9–12		Week 13–19		Week 20–26	
Admission Source (N=182,106)	N	%	N	%	N	%	N	%
Total Admissions	8,173.6	100.0	6,009.0	100.0	5,796.6	100.0	7,443.6	100.0
Home	7,613.0	93.1	5,572.1	92.7	5,413.9	93.4	6,969.2	93.7
Another Hospital	340.1	4.2	277.8	4.6	255.4	4.4	307.3	4.1
RCF	209.1	2.6	149.8	2.5	118.4	2.0	158.4	2.1
Other	11.4	0.1	9.3	0.2	8.9	0.2	8.7	0.1
χ2 (p-value)	-	-	-	1.90 (0.593)	-	4.52 (0.211)	-	3.29 (0.348)
Discharge Destination (N=180,664 *)	N	%	N	%	N	%	N	%
Total Discharges	8,108.3	100.0	5,963.3	100.0	5,743.2	100.0	7,391.8	100.0
Home	6,882.8	84.9	5,066.8	85.0	4,849.1	84.4	6,339.0	85.8
RCF	524.0	6.4	314.0	5.3	233.0	4.1	351.1	4.8
Died	200.3	2.5	148.5	2.5	144.2	2.5	147.4	2.0
Another Hospital	415.6	5.1	353.5	5.9	422.9	7.4	439.7	5.9
Other	85.6	1.1	80.5	1.3	94.0	1.6	114.6	1.5
χ2 (p-value)	-	-	-	14.93 (0.005)	-	72.01 (<0.0001)	-	36.40 (<0.0001)
Discharge Outcome (N=182,106)	N	%	N	%	N	%	N	%
Total Discharges	8,173.6	100.0	6,009.0	100.0	5,796.6	100.0	7,443.6	100.0
Dead	200.2	2.4	148.5	2.5	144.3	2.5	147.4	2.0
Alive	7,973.4	97.6	5,860.5	97.5	5,652.3	97.5	7,296.2	98.0
χ2 (p-value)	-	-	-	0.02 (0.903)	-	0.02 (0.888)	-	3.99 (0.05)
Gender (N=182,106)	N	%	N	%	N	%	N	%
Total Admissions	8,173.6	100.0	6,009.0	100.0	5,796.6	100.0	7,443.6	100.0
Female	4,073.6	49.8	2,896.8	48.2	2,792.9	48.2	3,641.3	48.9
Male	4,100	50.2	3,112.2	51.8	3003.7	51.8	3,802.3	51.1
χ2 (p-value)	-	-	-	3.68 (0.055)	-	3.74 (0.053)	-	1.33 (0.249)
CCI (N=182,106)	N	%	N	%	N	%	N	%
Total Admissions	8,173.6	100.0	6,009.0	100.0	5,796.6	100.0	7,443.6	100.0
<1	6,152.7	75.3	4,579.3	76.2	4,310.3	74.3	5,575.2	74.9
1–3	534.5	6.5	391.9	6.5	406.4	7.0	527.9	7.1
4–6	232.0	2.9	171.3	2.9	155.1	2.7	187.6	2.5
7–9	297.0	3.6	204.5	3.4	219.1	3.8	271.0	3.6
10+	957.4	11.7	662.0	11.0	705.7	12.2	881.9	11.9
χ2 (p-value)	-	-	-	2.33 (0.676)	-	2.60 (0.627)	-	3.28 (0.513)
Age Group (N=182,106)	N	%	N	%	N	%	N	%
Total	8,173.6	100.0	6,009.0	100.0	5,796.6	100.0	7,443.6	100.0
Age 0–14	948.8	11.6	689.3	11.5	491.6	8.5	594.4	8.0
Age 15–44	1,775.1	21.7	1,359.0	22.6	1,202.4	20.8	1,573.7	21.1
Age 45–64	1,905.1	23.3	1,438.2	23.9	1,508.4	26.0	1,882.1	25.3
Age 65–79	2,099.5	25.7	1,534.2	25.5	1,595.9	27.5	2,034.5	27.3
Age 80+	1,445.1	17.7	988.3	16.5	998.3	17.2	1,358.9	18.3
χ2 (p-value)	-	-	-	5.03 (0.284)	-	48.54 (<0.0001)	-	63.49 (<0.0001)

*N=1,142 missing for discharge destination and discharge outcome variables

In Period 4, HIPE analysis found that the rates of non-COVID-19 hospital admission remained below expected levels for all age groups compared to Period 1 (Rate Ratio 0.72, 95% CI 0.71, 0.73, p-value <0.0001) ( Figure 7). There was less recovery for elective admissions (Rate Ratio 0.63, 95% CI 0.62, 0.64, p-value <0.0001) compared to emergency admissions (Rate Ratio 0.90, 95% CI 0.87, 0.93, p-value <0.0001). During Period 4, there were increases in emergency mental health admissions (Rate Ratio 1.28, 95% CI 1.03, 1.60, p-value 0.028) ( Table 7), emergency alcohol-related admissions (Rate Ratio 1.22, 95% CI 1.03, 1.43, p-value 0.016) and emergency admissions with self-harm (Rate Ratio 1.39, 95% CI 1.01, 1.91, p-value 0.043) ( Table 8).

## Discussion

### Summary of key findings

This study reports on the changes in healthcare utilisation in acute hospitals in Ireland during the first wave of the COVID-19 pandemic in 2020. There was reduced healthcare utilisation for elective and emergency acute public hospital care. This reduction began in early March 2020, following the beginning of the first wave (Period 2), and overall persisted for the duration of this study which included the time period when society reopened and public health restrictions were eased; up to 5 ^th^ July 2020 (Period 4). During this period, population rates of elective non-COVID-19 care did not recover. In contrast, there was greater recovery of emergency healthcare utilisation rates, however, activity still remained below pre-COVID-19 levels, particularly among younger age groups. In particular, this study finds evidence of increased emergency alcohol and emergency mental health related admissions following the reopening of society (Period 4) which began on 18 ^th^ May 2020,

Those who presented to ED during the first wave of the COVID-19 pandemic had an increased likelihood of admission which may suggest increased severity of illness ^27^. However, this finding may be explained by reverse association and there is no evidence of an immediate increase in in-hospital mortality or an increase in co-morbidity on discharge. The full consequences of the impact of changes due to delayed or missed care on population health may only become apparent over time.

### Comparison with other studies

The findings of this study are consistent with other published reports and literature describing disruption to healthcare services during the first wave of the COVID-19 pandemic in multiple countries ^5, 31– 33^. In particular, reductions in ED presentations and hospital admissions which persisted following the easing of restrictions are reported ^32– 34^. While a proportion of these reductions were likely due to decreased incidence of certain conditions related to population-level restrictions, some necessary care was not accessed for acute medical emergencies (e.g., stroke and AMI) ^3– 5, 32, 35^. The greatest reductions in presentations were reported among vulnerable groups such as lower socioeconomic groups, those at extremes of age and ethnic minorities ^5, 36^.

This study found no evidence of immediate harm related to delayed or lost presentations. This is in contrast to other studies, which reported evidence of increased morbidity and mortality associated with changes in healthcare utilisation ^4, 5, 37^. This impact may only become fully apparent over time and through examination of wider health information datasets.

### Reasons for changes in acute hospital utilisation

The reasons for the changes in healthcare utilisation during the study period are likely multifactorial. The COVID-19 pandemic highlighted well-established weaknesses in the Irish health system that pre-date the COVID-19 pandemic. These include the absence of universal healthcare, acute hospital capacity deficits and a service configuration with overreliance on the acute hospital system to provide scheduled as well as unscheduled care due to poor orientation to primary and community care ^7, 10, 11, 38, 39^. In order to create capacity to manage acute COVID-19 and non-COVID-19 illness, it was necessary to postpone elective care in the acute hospitals ^38^. While some time-critical elective care was diverted to private hospitals ^40^, analysis of clinical patterns of elective admissions in this study suggest that there are large backlogs in care. This finding is supported by the increases in waiting lists for elective care that occurred during this time period, suggesting that the demand for elective care was not fully met by the private sector ^41^. In the period following the first wave of the COVID-19 pandemic in 2020, when society reopened, ongoing capacity restrictions in healthcare settings and the need to provide care for those with COVID-19 infection meant that it was not possible to resume elective activity at pre-COVID-19 levels or to provide the level of services required to fully address backlogs in elective care ^42, 43^. For emergency care, the reduction may have been due to reduced incidence of some medical conditions, e.g., injuries and non-COVID-19 infections, due to population-level restrictions and/or due to a reduction in unnecessary emergency attendances ^26, 35, 44– 49^. However, the scale of the reductions shown in this study and reduction in presentations for conditions such as stroke/TIA and AMI which are non-discretionary and time-sensitive suggest that necessary care was avoided or delayed. This may have been due to a fear of exposure to COVID-19 in hospital ^50, 51^. Deaths due to untreated acute medical emergencies may have also contributed to the lack of ‘rebound’ admissions observed following the reopening of society, Increased utilisation of acute health services for emergency alcohol and self-harm admissions in the period immediately following wave 1 suggests that the pandemic, and associated restrictions, are negatively impacting population health and wellbeing. This finding is consistent with published data reporting increased mental distress and increased utilisation of secondary mental health services due to the COVID-19 pandemic ^52– 55^. This burden of unmet need is likely greatest among vulnerable groups most affected by COVID-19 such as those living in poverty, ethnic minority groups and older people ^2, 24, 56– 58^.

### Implications for health policy and health system reform in Ireland

***Harnessing the COVID-19 shock to manifest health system change.*** COVID-19 is a shock to the health system ^59^. However, despite the challenges, the system has responded and shown innovation and flexibility in work practices and delivery of services, which demonstrate capacity and readiness to reform ^11, 38^. Lessons must be learned from COVID-19 to build health system resilience and increase preparedness for the future, including future pandemic preparedness ^59, 60^. In the long-term, further strategic reform aligned with Sláintecare should be progressed building on this innovation and change capability shown during the COVID-19 pandemic. Internationally, there have been calls to ‘build back better’ and also to ‘build back fairer’ to achieve sustainable, resilient health systems and deliver universal healthcare. Such an endeavour will require political leadership, human and financial resources and investment in information technology (IT) infrastructure and public health expertise ^11, 61– 65^. In Ireland, this will need to include addressing now well-acknowledged public health service capacity investment deficits ^66^, which were partially relieved on a temporary basis through bespoke pandemic-specific arrangements with private hospitals. The findings of this study were disseminated nationally to the director of Sláintecare and to the national leads for Integrated Care, the Acute Hospitals and Mental Health. This study quantified the changes in healthcare utilisation during the first wave of the COVID-19 pandemic and identified key clinical areas to focus on for population health recovery. The findings are important in the context of the ongoing reform of the Irish health system and the findings of this study informed the HSE National Service Plan for 2022 which has a focus on scheduled care recovery ^67^.

***Public health should be core to health reform.*** Public health has been frontline in confronting initial waves of COVID-19 in Ireland. With the development and arrival of the COVID-19 vaccination in 2021, Ireland has entered a new phase of the COVID-19 pandemic. However, COVID-19 and its associated consequences will continue to impact population health and the health system for many years. Therefore, strong public health leadership and advocacy are required to seize the opportunity to control COVID-19 infection, to guide population health recovery from COVID-19 and to progress health system reform in Ireland

### Limitations of this study

Due to the data available at the time of analysis, this study focuses only on the first wave of COVID-19. While the patterns observed in this study may predict healthcare utilisation in subsequent waves, there are likely differences as some lessons learned from the first wave may have been acted on. PET and HIPE datasets do not allow for identification of repeat episodes of care which may overestimate population rates of healthcare utilisation. However, such an overestimate is likely to be minimal due to the large size of the datasets. During the first wave of the COVID-19 pandemic, some time-sensitive elective care was provided in the private hospitals, these data were not available for this study. Therefore, the reduction in elective hospital activity may be overestimated. Data on GP utilisation were not analysed in this study, changes in provision of GP care may explain some of the changes reported. Hospital outpatient department (OPD) activity was also not examined, this may underestimate need for services as there are backlogs for OPD appointments. PET does not contain clinical information therefore the impact on non-COVID-19 care was not quantified. HIPE reports data on patients discharged from acute hospitals. Therefore, patients who remained in hospital at the end of the study period are not included in this study. As those who are more unwell may have longer admissions with poorer outcomes, co-morbidity and in-hospital mortality may have been underestimated. 

## Conclusion

This study quantifies and describes changes in acute hospital care utilisation during the first wave of the COVID-19 pandemic in Ireland. The results show that there are large backlogs in elective care, and evidence of delayed and lost emergency care. These backlogs in care must be managed with urgency. The consequences of delayed and lost care will only become fully apparent over time. The results also demonstrate increased population need and demand for mental health and alcohol services triggered by the pandemic. The population health impacts of COVID-19 and associated restrictions, particularly in relation to mental health and alcohol, need to be addressed through strong public health and health systems responses including the adoption of a pandemic recovery plan, especially targeting the most vulnerable. COVID-19 highlights inherent weakness in the Irish health system. However, the system shock is an opportunity to progress strategic reform of the Irish health system towards a universal, high-quality, sustainable and resilient health system, capable of meeting population health needs and responding to future pandemics.

## Data Availability

Open Science Framework. The public health and health system implications of changes in the utilisation of acute hospital care in Ireland during the first wave of COVID-19: Lessons for recovery planning. DOI: https://doi.org/10.17605/OSF.IO/D56SZ ^68^ This project contains the following underlying data: -   The public health and health system implications of changes in the utilisation of acute hospital care_Supplementary Tables.pdf -   The public health and health system implications of changes in the utilisation of acute hospital care_RECORD Checklist.pdf Data are available under the terms of the Creative Commons Attribution 4.0 International license The datasets processed for this study were derived from special categories of personal data concerning health. The datasets are controlled by the HSE, not the authors, and so the authors cannot determine requests for data access. Further information on HSE data protection policy can be located at hse.ie/eng/gdpr/hse-data-protection-policy/. Reasonable requests to access the two datasets used in this study, HIPE and PET, can be directed to the data controller by contacting the HSE Healthcare Pricing Office ( https://hpo.ie/) in the case of HIPE and to the HSE Special Delivery Unit https://www.hse.ie/eng/about/who/acute-hospitals-division/special-delivery-unit/ in the case of PET. OSF registries. RECORD guideline checklist, extended from the STROBE statement. DOI: https://doi.org/10.17605/OSF.IO/D56SZ ^68^ Data are available under the terms of the Creative Commons Attribution 4.0 International license (CC-BY 4.0).
