# Supplementary material for: Changes in the utilisation of acute hospital care in Ireland during the first wave of the COVID-19 pandemic in 2020
Source: HRB Open Res. 2022 Oct 12;4:67. Originally published 2021 Jun 23. [Version 3] doi: 10.12688/hrbopenres.13307.3 (PMC9513415; doi:10.12688/hrbopenres.13307.3)
Supplement: Peer review history

# Peer review: Reviewer response for version 3

**DOI:** 10.21956/hrbopenres.14914.r33021

**Published:** 2022-10-13

**Authors:** Akke Vellinga

**Affiliations:** 1 School of Public Health, Physiotherapy and Sports Science, University College Dublin, Dublin, Ireland

I am satisfied with the changes.

Is the work clearly and accurately presented and does it cite the current literature?

Yes

If applicable, is the statistical analysis and its interpretation appropriate?

Partly

Are all the source data underlying the results available to ensure full reproducibility?

Yes

Is the study design appropriate and is the work technically sound?

Yes

Are the conclusions drawn adequately supported by the results?

Partly

Are sufficient details of methods and analysis provided to allow replication by others?

Yes

Reviewer Expertise:

Epidemiology, infectious disease, general practice, statistics, prescribing, antibiotics, AMR, data, data dashboards

I confirm that I have read this submission and believe that I have an appropriate level of expertise to confirm that it is of an acceptable scientific standard.

# Peer review: Reviewer response for version 2

**DOI:** 10.21956/hrbopenres.14826.r32823

**Published:** 2022-09-26

**Authors:** Akke Vellinga

**Affiliations:** 1 School of Public Health, Physiotherapy and Sports Science, University College Dublin, Dublin, Ireland

This is an interesting study showing some of the impacts of the pandemic on the Irish Health System. Whereas the authors have already addressed many issues raised by the other reviewers, an overall lack of context remains. The data sets used are from public hospitals only. One positive outcome of the pandemic was the use of private hospitals. They took over much of the elective and non-covid hospital care from the public hospitals. Whereas there is a mention in the limitations of the study, it should be better reflected in the whole manuscript. Reductions in non-covid admissions in the data may just be because these admissions happened in the private hospitals, not necessarily because they did not happen. This should be discussed in the results. It is now presented as if there just is a reduction due to the pandemic, which is true, but not the whole story. The reduction of 35% in non-covid admissions may be entirely explained by redirecting these to private hospitals. 

 The term recovery period is not appropriate. By July 2020 there was no recovery, in either healthcare use, the pandemic or otherwise. Please choose a better term throughout (or just stick with period 4).

 Admission to hospitals changed radically during the pandemic, and most likely those who were sicker were more likely to be admitted. These people were sicker in general when they presented and therefore more likely to be admitted. What was not clear to me is if the fewer admissions were compared to the same period the previous year (which would be because of less presentations) or if this is a subset of the presentations. I suspect it is the first. If so, the figures should present the admissions as a subset of the total presentations, which could show that admissions from ED are actually higher.

 The tracer conditions are of interest, as also pointed out by the other reviewers. In particular their rise in period 4. Whereas this is an interesting paper, the focus of the discussion is only on the reductions during period 2-3, while the tracer conditions and the potential of the health system to use private hospitals as part of a public system seem to be the main lessons from this paper/the pandemic. I would suggest refocusing the discussion.

 The expected 'rebound' of the admissions in period 4 may not be seen due to death from covid or the condition for which they did not present (AMI, cancer, other). This should be discussed.

 Some of the variables showing significance in the logistic regression can also be explained by reverse association. Please add to the discussion.

Is the work clearly and accurately presented and does it cite the current literature?

Yes

If applicable, is the statistical analysis and its interpretation appropriate?

Partly

Are all the source data underlying the results available to ensure full reproducibility?

Yes

Is the study design appropriate and is the work technically sound?

Yes

Are the conclusions drawn adequately supported by the results?

Partly

Are sufficient details of methods and analysis provided to allow replication by others?

Yes

Reviewer Expertise:

Epidemiology, infectious disease, general practice, statistics, prescribing, antibiotics, AMR, data, data dashboards

I confirm that I have read this submission and believe that I have an appropriate level of expertise to confirm that it is of an acceptable scientific standard, however I have significant reservations, as outlined above.

# Response

**Published:** 2022-10-04

**Authors:** Louise Marron

**Affiliations:** Health Service Executive , Ireland

**Comment 1:** This is an interesting study showing some of the impacts of the pandemic on the Irish Health System. Whereas the authors have already addressed many issues raised by the other reviewers, an overall lack of context remains. The data sets used are from public hospitals only. One positive outcome of the pandemic was the use of private hospitals. They took over much of the elective and non-covid hospital care from the public hospitals. Whereas there is a mention in the limitations of the study, it should be better reflected in the whole manuscript. Reductions in non-covid admissions in the data may just be because these admissions happened in the private hospitals, not necessarily because they did not happen. This should be discussed in the results. It is now presented as if there just is a reduction due to the pandemic, which is true, but not the whole story. The reduction of 35% in non-covid admissions may be entirely explained by redirecting these to private hospitals. 

**Reply 1:** Many thanks for this comment. This study examined healthcare utilisation in public hospitals. Data on private hospital activity during the first wave of the COVID-19 pandemic were not available for this study. This has now been stated more clearly in the methods. In the limitations, this paper also states that *during the first wave of the COVID-19 pandemic, some time-sensitive elective care was provided in the private hospitals, these data were not available for this study*. *Therefore, the reduction in elective hospital activity may be overestimated*.

 During 2020, waiting lists for elective care increased despite private hospital activity and during the time period for this study in 2020, the National Public Health Emergency Team (NPHET) recommended the cancellation of non-essential care. There is no evidence suggesting that all the need for elective care was met in the private sector during the first wave of COVID-19 and this has been evident from the increase in waiting lists for elective care nationally. This is now stated more clearly in the discussion with an additional reference added for a recently published paper by Mercille et al., 2022. 

**Comment 2: **The term recovery period is not appropriate. By July 2020 there was no recovery, in either healthcare use, the pandemic or otherwise. Please choose a better term throughout (or just stick with period 4).

**Reply 2: **Many thanks for your comment about the use of the term recovery period for Period 4. The rationale for the study time periods is outlined in Table 1. The time periods were defined based on the epidemiology of COVID-19 and the recommended public health measures during the study time period. The term recovery period for Period 4 was used to reflect the reopening of society and the easing of public health restrictions and the significant reduction in notified COVID-19 cases during this time period. When this study was conducted in 2020, the future course of the COVID-19 pandemic was unknown. The term recovery period has now been replaced with the terms reopening of society, easing of public health restrictions and Period 4 in the paper.

**Comment 3:** Admission to hospitals changed radically during the pandemic, and most likely those who were sicker were more likely to be admitted. These people were sicker in general when they presented and therefore more likely to be admitted. What was not clear to me is if the fewer admissions were compared to the same period the previous year (which would be because of less presentations) or if this is a subset of the presentations. I suspect it is the first. If so, the figures should present the admissions as a subset of the total presentations, which could show that admissions from ED are actually higher

**Reply 3: **Thank you for this comment. This paper presents the population rates of Emergency Department (ED) presentations, admissions from ED and the population rates of non-COVID-19 hospital admission (elective and emergency) using two datasets; PET and HIPE As part of the methodology, two reference periods were used; the same time periods in 2019 and also Period 1 of 2020 (pre-COVID-19) to compare population healthcare utilisation trends during pre- and post-COVID-19 time periods. Population rates for each outcome were calculated using Census 2016 as the denominator. The changes in the trends in healthcare utilisation including hospital admission following presentation to ED and non-COVID-19 hospital admissions were the same overall for each reference period. Data from the internal comparison within 2020 are presented in this paper. It is clear from this analysis that overall, the population rates of emergency admissions reduced comparing pre- and post-COVID-19 time periods. The results presented show the findings from the analysis that aligns with the study aims and objectives and methodology used to describe changes in healthcare utilisation during the first wave of the COVID-19 pandemic in Ireland.

**Comment 4**: The tracer conditions are of interest, as also pointed out by the other reviewers. In particular their rise in period 4. Whereas this is an interesting paper, the focus of the discussion is only on the reductions during period 2-3, while the tracer conditions and the potential of the health system to use private hospitals as part of a public system seem to be the main lessons from this paper/the pandemic. I would suggest refocusing the discussion.

**Reply 4: **Many thanks for this comment about the tracer conditions. The significant increases in emergency admissions for self-harm, alcohol and acute mental health in Period 4 were key findings of this study and this is emphasised in the discussion. The reduction in key tracer conditions such as stroke and acute myocardial infarction are were also significant findings from a clinical and public health perspective and are included in the discussion. The section in the discussion *Harnessing the COVID-19 shock to manifest health system change *has been expanded to include more discussion about the role of the private hospitals. The Irish Government Economic and Evaluation Service Spending Review 2021 has been included as a reference in addition to the other reference also added to the discussion from Mercille *et al.,* 2022. 

**Comment 5:** The expected 'rebound' of the admissions in period 4 may not be seen due to death from covid or the condition for which they did not present (AMI, cancer, other). This should be discussed.

**Reply 5:** Thank you for this comment that rebound admissions may not be seen due to deaths from COVID-19 or from delayed/lost emergency care. This has been added to the discussion. 

**Comment 6 **Some of the variables showing significance in the logistic regression can also be explained by reverse association. Please add to the discussion.

**Reply 6: **Many thanks for this comment about reverse association. This has been added to the paper.

 Many thanks for taking the time to review this paper and for your comments.

# Peer review: Reviewer response for version 2

**DOI:** 10.21956/hrbopenres.14826.r32279

**Published:** 2022-06-23

**Authors:** Therese McDonnell

**Affiliations:** 1 IRIS Centre, School of Nursing, Midwifery and Health Systems, University College Dublin, Dublin, Ireland

Thank you for the revisions made in response to my comments. The title and methods now better reflect the content of the paper. Other alterations noted.

Is the work clearly and accurately presented and does it cite the current literature?

Yes

If applicable, is the statistical analysis and its interpretation appropriate?

Yes

Are all the source data underlying the results available to ensure full reproducibility?

Partly

Is the study design appropriate and is the work technically sound?

Yes

Are the conclusions drawn adequately supported by the results?

Partly

Are sufficient details of methods and analysis provided to allow replication by others?

Yes

Reviewer Expertise:

Health economics

I confirm that I have read this submission and believe that I have an appropriate level of expertise to confirm that it is of an acceptable scientific standard.

# Peer review: Reviewer response for version 1

**DOI:** 10.21956/hrbopenres.14487.r31956

**Published:** 2022-05-12

**Authors:** Therese McDonnell

**Affiliations:** 1 IRIS Centre, School of Nursing, Midwifery and Health Systems, University College Dublin, Dublin, Ireland

This is an interesting paper using high quality national data on ED attendance and admissions at public hospitals in Ireland to identify how attendance/admissions altered over the initial weeks of the onset of COVID-19 in 2020.

**Specific comments:**

- There is mention within the paper and in the abstract of interrupted time-trend analysis.  I don’t see this in the Results, so this reference needs to be amended.
- Table 1: the date range for Period 4 needs to be amended.
- Selecting the tracer diagnoses is valuable, perhaps this can be better motivated in the *Outcomes *section.
- The logistic regression (Table 6) gives little insight on how predictors altered the likelihood of admission after Period 1.  Consider re-focusing this regression – perhaps through this use of interactions, comparison with prior years, or a multinomial logit.
- I think the discussion needs to better reflect the results. The most impactful finding of this paper is the lack of a rebound in hospital admissions for elective procedures in Period 4. While this is discussed in the *Reasons for change in acute hospital utilisation* section, it should also be clearly mentioned in the first paragraph of the Discussion: *Summary of findings.* This first paragraph also makes a statement suggesting evidence of increased severity in Periods 2 - 4, however the triage classifications in the ED tables suggest little change in severity. Ambulance usage may have increased due to the restrictions on movement (lack of transport) and due to some individuals seeking guidance from paramedics on the need to attend hospital. Age may not be directly related to severity, an increase in the age of admissions may also be linked to a greater usage of ambulance. The *Reasons for change in acute hospital utilisation* section notes COVID-19 has highlighted the weaknesses in the Irish health system, and a number of references are given.  I would recommend reviewing these references – a number pre-date COVID-19 and not all support that COVID-19 highlighted the shortcomings of the system.  Perhaps rewording is needed here.
- I would suggest reframing the title to match what the paper actually achieved.  I don’t feel the paper delivers on the title as currently stated, and I’m unsure what lessons derive directly from this analysis, for example I don't see the relationship with universal healthcare.
- Overall, I think this is a good paper that presents a national picture that makes a valuable contribution to the literature.

Is the work clearly and accurately presented and does it cite the current literature?

Yes

If applicable, is the statistical analysis and its interpretation appropriate?

Yes

Are all the source data underlying the results available to ensure full reproducibility?

Partly

Is the study design appropriate and is the work technically sound?

Yes

Are the conclusions drawn adequately supported by the results?

Partly

Are sufficient details of methods and analysis provided to allow replication by others?

Yes

Reviewer Expertise:

Health economics

I confirm that I have read this submission and believe that I have an appropriate level of expertise to confirm that it is of an acceptable scientific standard, however I have significant reservations, as outlined above.

# Response

**Published:** 2022-05-30

**Authors:** Louise Marron

**Affiliations:** Health Service Executive , Ireland

Many thanks for your comments and feedback and for taking the time to review this paper.

**Comment 1:**

 There is mention within the paper and in the abstract of interrupted time-trend analysis. I don’t see this in the results so this reference needs to be amended.

**Reply 1:**

 Thank you for your comment. This study was a comparison of population rates of healthcare utilisation during different time periods in the first wave of COVID-19 infection in Ireland in 2020; the time periods were defined based on the epidemiology of the first wave and the associated population level restrictions. It was described in the paper as an interrupted time-trend analysis. I have removed this term from the paper and abstract and I have updated the manuscript and described the study as a retrospective, population-based observational study which more accurately reflects the methodology.

**Comment 2:**

 Table 1: the date range for Period 4 needs to be amended.

**Reply 2:**

 Many thanks for flagging this error. I have amended this.

**Comment 3:**

 Selecting the tracer diagnoses is valuable, perhaps this can be better motivated in the Outcomes section.

**Reply 3:**

 Many thanks for this comment. I have added further information and detail to the outcomes section further explaining the purpose of selecting the tracer conditions, how the tracer conditions were selected and the rationale for their selection.

**Comment 4:**

 The logistic regression (Table 6) gives little insight on how predictors altered the likelihood of admission after Period 1. Consider re-focusing this regression – perhaps through the use of interactions, comparison with prior years or a multinomial logit.

**Reply 4:**

 Thank you for your comment. The purpose of the binary logistic regression analysis was to assess if presentation to ED within the specific time periods in the study was associated with an increased likelihood of admission from ED. The main finding was that independent of all other factors collected as part of PET data that may influence admission, there was an increased likelihood of being admitted to hospital following ED presentation in Periods 2–4 compared to Period 1, which was most marked in Period 3 (the peak of restrictions). Increased admissions from ED may suggest increased severity of illness on presentation (I have included a reference for this) and this was also in keeping with the clinical experience reported to the authors by clinicians at the time. I have edited the methods and the findings to more accurately reflect the purpose of the regression which has a limited impact on the overall findings of this paper.

**Comment 5:**

 I think the discussion needs to better reflect the results. The most impactful finding of this paper is the lack of a rebound in hospital admissions for elective procedures in Period 4. While this is discussed in the *Reasons for change in acute hospitalisation* section, it should also be clearly mentioned in the first paragraph of the *Discussion: Summary of findings*. This first paragraph also makes a statement suggesting evidence of increased severity on Periods 2-4, however the triage classifications in the ED tables suggest little change in severity. Ambulance usage may have increased due to the restrictions on movement (lack of transport) and due to some individuals seeking guidance from paramedics on the need to attend hospital. Age may not be directly related to severity, an increase in the age of admissions may also be linked to a greater usage of ambulance. The Reasons for change in healthcare utilisation section notes that COVID-19 has highlighted the weaknesses in the Irish health system, and a number of references are given. I would recommend reviewing these references – a number pre-date COVID-19 and not all support that COVID-19 highlighted the shortcomings of the system. Perhaps rewording is needed here.

**Reply 5:**

 Thank you for your comments and insights into the findings of this paper. I have added that in particular, rates of elective non-COVID-19 care did not recover following the first wave to the *summary of key findings section* in the discussion. I have also discussed this further in the *reasons for changes in acute hospital utilisation *section. Specifically, I have noted that this lack of recovery in elective care was due to ongoing capacity restrictions in healthcare settings and the ongoing need to direct resources to the provision of COVID-19 care. I have referenced the 2020 Winter Plan and the 2021 HSE service plan. In the summary of key findings section, I have taken out mention of the greater proportion that arrived by ambulance and the older age groups as findings which may suggest increased severity of illness. I have noted that there was increased likelihood of admission from ED which may suggest increased severity of illness and noted no evidence of increase in in-hospital mortality or increase in co-morbidity on discharge. I have reworded the *reasons for changes in acute hospital utilisation section *to more clearly make the point that the weakness in the Irish health system that I mention and reference did pre-date COVID-19 and as a result these weaknesses impacted the COVID-19 response.

**Comment 6:**

 I would suggest reframing the title to match what the paper actually achieved. I don’t feel the paper delivers on the title as currently stated, and I’m unsure what lessons derive directly from this analysis, for example I don’t see the relationship with universal healthcare.

**Reply 6:**

 Many thanks for your comment. I have changed the title of the paper to Changes in the utilisation of acute hospital care in Ireland during the first wave of the COVID-19 pandemic in 2020 which is an accurate reflection of what the paper reports.

**Comment 7:**

 Overall, I think this is a good paper that presents a national picture that makes a valuable contribution to the literature

**Reply 7:**

 Many thanks for your comments and feedback and for taking the time to review this paper.

# Peer review: Reviewer response for version 1

**DOI:** 10.21956/hrbopenres.14487.r30817

**Published:** 2021-12-13

**Authors:** Kednapa Thavorn

**Affiliations:** 1 Clinical Epidemiology Program, Ottawa Hospital Research Institute, Ottawa, ON, Canada

This population-based study described the utilization of acute hospital services during the first wave of the COVID-19 pandemic in Ireland. The study was based on two national databases; ED attendances were obtained from Patient Experience Time (PET), while acute hospital discharge data were derived from Hospital In-Patient Enquiry (HIPE). Descriptive statistics and regression analysis were used to compare presentation to and admission from ED and acute hospital admission of any type for a non-COVID-19 illness across the four study periods, representing the epidemiology and public health management of COVID-19 in Ireland. This study shows that the first wave of COVID-19 was associated with decreased ED presentations, fewer admissions from ED, and non-COVID-19 hospital admissions.

- The study's title does not directly align with the study findings and is misleading. The study assessed the impact of the pandemic and the public health and health system measures on health care utilization; it did not fully describe how changes in health care utilization may affect public health or the health care system.
- There are some inconsistencies across the manuscript. In the abstract, the authors indicated that they used an interrupted time-series (ITS) analysis, but this methodology was not mentioned or used in the main texts. Instead, logistic regression was used. Please verify and ensure consistency. Additionally, the results section compared outcomes between four exposure periods and historical periods. The historical period was not defined or described in the method section. It is unclear why the first period (06/01/2020–01/03/2020) and the historical period (2017-19 or 2019) were used interchangeably as a reference group.
- What was the purpose of the inclusion of tracer diagnoses? Please provide further descriptions.
- The result section provides primarily descriptive results, which are difficult to interpret due to the potential impact of seasonal and trend patterns. If the authors did not use an ITS, they should focus on the results of multiple logistic regression analyses, as this method helped adjust for some confounding factors. The adjusted results would be more informative and more appropriate to compare against the existing literature.  
- How were Figures 3 – 7 generated? Were they based on multiple regression analyses? If they were, shouldn't the y-axis be shown as adjusted odd ratios instead of rate ratios? There were three study outcomes? Why were only the regression results of ED admission reported in the manuscript (Table 6)?
- The discussion on health policy and health system implications was too broad; it is unclear how the study findings led to these policy recommendations.

Is the work clearly and accurately presented and does it cite the current literature?

Yes

If applicable, is the statistical analysis and its interpretation appropriate?

Partly

Are all the source data underlying the results available to ensure full reproducibility?

Partly

Is the study design appropriate and is the work technically sound?

Partly

Are the conclusions drawn adequately supported by the results?

Partly

Are sufficient details of methods and analysis provided to allow replication by others?

No

Reviewer Expertise:

Health services research

I confirm that I have read this submission and believe that I have an appropriate level of expertise to confirm that it is of an acceptable scientific standard, however I have significant reservations, as outlined above.

# Response

**Published:** 2022-05-30

**Authors:** Louise Marron

**Affiliations:** Health Service Executive , Ireland

Many thanks for your comments and feedback and for taking the time to review this paper.

**Comment 1:**

 The study's title does not directly align with the study findings and is misleading. The study assessed the impact of the pandemic and the public health and health system measures on health care utilization; it did not fully describe how changes in health care utilization may affect public health or the health care system.

**Reply 1:**

 Thank you for your comment, I have changed the title of the paper to 'Changes in the utilisation of acute hospital care in Ireland during the first wave of the COVID-19 pandemic in 2020' which is an accurate reflection of what the study reports.

**Comment 2:**

 There are some inconsistencies across the manuscript. In the abstract, the authors indicated that they used an interrupted time-series (ITS) analysis, but this methodology was not mentioned or used in the main texts. Instead, logistic regression was used. Please verify and ensure consistency. Additionally, the results section compared outcomes between four exposure periods and historical periods. The historical period was not defined or described in the method section. It is unclear why the first period (06/01/2020–01/03/2020) and the historical period (2017-19 or 2019) were used interchangeably as a reference group.

**Reply 2:**

 Thank you for your comment. This was not an interrupted time series analysis and this term was not used in the manuscript. This study was a comparison of population rates of healthcare utilisation during different time periods in the first wave of COVID-19 infection in Ireland in 2020; the time periods were defined based on the epidemiology of the first wave and the associated population level restrictions. It was described in the paper as an interrupted time-trend analysis. On reflection, I agree that the term is unclear. Therefore, I have removed this term and I have described the study as a retrospective, population-based observational study which more accurately reflects the methodology. Thank you also for your comment regarding the need for more clarity about the historical reference periods and the reference period within 2020. I have included further details about this in the methodology and results sections. I have defined the time periods in the methods section and explained that both reference periods were used and the trends and associated public health implications were found to be similar so the paper primarily focuses on the internal comparison within 2020. The two comparisons showing similar trends also suggests that the variation observed within 2020 was not related only to seasonal differences in healthcare utilisation trends.

**Comment 3:**

 What was the purpose of the inclusion of tracer diagnoses? Please provide further descriptions

**Reply 3:**

 Thank you for your comment. I have provided further detail in the updated manuscript. The purpose of selecting the ‘tracer diagnoses’ was to explore healthcare utilisation trends in key clinical areas where changes in healthcare utilisation had been observed. These conditions were chosen following a review of the literature and from discussions with the national HSE Lead for Integrated Care, the Lead for the Acute Hospitals and for Mental Health who were clinicians providing frontline care during the COVID-19 pandemic in Ireland. Inclusion of the tracer diagnoses allowed us to better delineate more specific implications for key clinical conditions to inform recovery planning. I have included further detail on this in the outcomes section of the methods section.

**Comment 4:**

 The result section provides primarily descriptive results, which are difficult to interpret due to the potential impact of seasonal and trend patterns. If the authors did not use an ITS, they should focus on the results of multiple logistic regression analyses, as this method helped adjust for some confounding factors. The adjusted results would be more informative and more appropriate to compare against the existing literature.

**Reply 4:**

 Thank you for your comment. The results primarily focus on the absolute and relative differences in rates of healthcare utilisation in pre and post COVID-19 time periods. The purpose of including two reference periods was to assess if population trends in healthcare utilisation were similar for both periods, the historic (pre-2020) comparison allowed delineation of seasonal effects. Unfortunately, the logistic regression analysis could only be carried out for PET data as the HIPE data are aggregate data. I have stated this more clearly in the methods and I have included further detail on the logistic regression analysis. As outlined in the data sources section of the paper, HIPE is a well-established, quality-assured health information system that is the primary source of episode-based clinical, demographic and administrative data on discharges from acute public hospitals in Ireland. HIPE data are aggregate data that are used nationally to inform healthcare planning, management and activity-based funding. For this study the HIPE data provided details of non-COVID-19 admissions to the acute hospitals, it identified changes in trends for all non-COVID-19 admissions and in particular changes for elective and emergency admissions and differences in the changes observed between elective and emergency admissions.

**Comment 5:**

 How were Figures 3 – 7 generated? Were they based on multiple regression analyses? If they were, shouldn't the y-axis be shown as adjusted odd ratios instead of rate ratios? There were three study outcomes? Why were only the regression results of ED admission reported in the manuscript (Table 6)?

**Reply 5:**

 Thank you for your comment. Figures 3 to 7 show the population rates of healthcare utilisation presented as weekly rates (Figures 3 and 4) and rate ratios (Figures 5, 6 and 7). These figures were not generated from regression analysis but from calculation of population rates of healthcare utilisation and comparison between pre- and post-COVID-19 time periods. I have clarified this further in the methods and the axes of the figures are labelled to reflect what is shown. There were three study outcomes, outcome was a non-COVID-19 hospital admission. This outcome is reported in the results, the rate difference and rate ratio per 100,000 population. However, as the reductions in non-COVID-19 hospital admissions were predominantly for elective and emergency admissions and as there were notable differences in the patterns and trends observed between elective and emergency admissions, these are presented separately in Figures 3, 4 5 and 7. I have clarified this in the results section. Regression analysis was only possible for PET data which are individual level data while the HIPE data are episode based and are aggregate data. I have updated the data sources section of the paper to more clearly state this. The purpose of the regression analysis was to identify factors, including time period, on the likelihood of admission from ED following presentation to ED. I have amended the methods section to more accurately reflect the purpose of the regression analysis reported in Table 6.

**Comment 6:**

 The discussion on health policy and health system implications was too broad; it is unclear how the study findings led to these policy recommendations.

**Reply 6:**

 Thank you for your comment. I have changed the title of the paper. I have also updated the discussion to include that the findings of this study were disseminated nationally to the director of Sláintecare and to the national leads for integrated care, the acute hospitals and mental health. I have added that this study quantified the changes in healthcare utilisation during the first wave of the COVID-19 pandemic and identified key clinical areas to focus on for population health recovery which are important in the context of the ongoing reform of the Irish health system. I have also added that the findings of this study informed the HSE National Service Plan for 2022 which has a focus on scheduled care recovery with an included reference to the plan.
